# Supplementary figures and images for: Antiviral and anti-inflammatory evaluation of herbal extracts: Implications for the management of calf diarrheal diseases
Source: PLoS One. 2026 Feb 6;21(2):e0342013. doi: 10.1371/journal.pone.0342013 (PMC12880661; doi:10.1371/journal.pone.0342013)

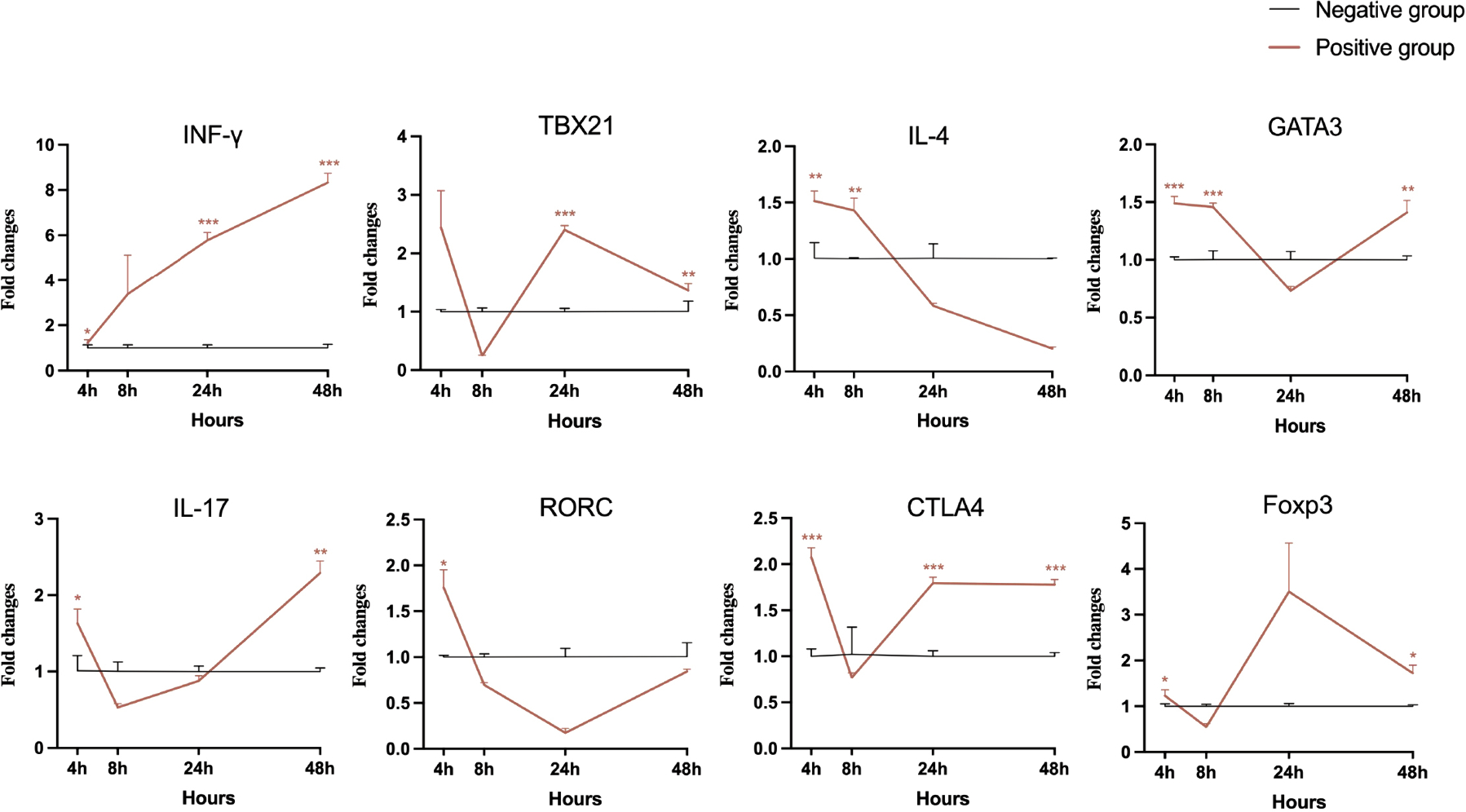

Supplement: S2 Fig — The positive control contained 1 μg/mL lipopolysaccharide derived from Escherichia coli. Asterisks (**p < 0.01, ***p < 0.001) indicate significant differences compared with the negative control group at the same time points. (TIF) [file pone.0342013.s002.tif]
