# Supplementary material for: Antiviral and anti-inflammatory evaluation of herbal extracts: Implications for the management of calf diarrheal diseases
Source: PLoS One. 2026 Feb 6;21(2):e0342013. doi: 10.1371/journal.pone.0342013 (PMC12880661; doi:10.1371/journal.pone.0342013)
Supplement: Attachment — Submitted filename: PONE-D-25-49741.pdf reviewed Oct. 2025.pdf [file pone.0342013.s004.pdf]

PLOS ONE

# Antiviral, anti-inflammatory and immunomodulatory effects of herbal extracts of Boswellia serrata, Commiphora myrrha, Scutellaria baicalensis, Gardenia jasminoides, Nypa fruticans and propolis

--Manuscript Draft--

| | |
|---|---|
| **Manuscript Number:** | PONE-D-25-49741 |
| **Article Type:** | Research Article |
| **Full Title:** | Antiviral, anti-inflammatory and immunomodulatory effects of herbal extracts of Boswellia serrata, Commiphora myrrha, Scutellaria baicalensis, Gardenia jasminoides, Nypa fruticans and propolis |
| **Short Title:** | Antiviral, anti-inflammatory and immunomodulatory effects of herbal extracts |
| **Corresponding Author:** | Han Sang Yoo<br>Seoul National University College of Veterinary Medicine<br>Seoul, KOREA, REPUBLIC OF |
| **Keywords:** | Herbal extracts; Bovine rotavirus; Bovine coronavirus; Antiviral activity; Anti-inflammatory effect; Immunomodulation |
| **Abstract:** | Traditional herbal extracts are attracting attention in the context of animal disease control because of their low side effects, diverse bioactive compounds, and low antimicrobial resistance risk. However, the underlying mechanisms remain inadequately understood. To characterize the multifaceted biological activities that underlie their therapeutic potential, this study systematically evaluated the antiviral, anti-inflammatory, and immunomodulatory properties of three herbal extracts in vitro. The antiviral activities of the extracts were tested against bovine rotavirus and bovine coronavirus, two major pathogens of neonatal calf diarrhea, and their anti-inflammatory effects were assessed in a lipopolysaccharide-stimulated murine macrophage model (RAW 264.7 cells) by measuring nitric oxide production and the expression of inflammation-related enzymes. Immunomodulatory effects were investigated by analyzing the gene expression of T helper cell-associated cytokines in both RAW 264.7 cells and bovine peripheral blood mononuclear cells (PBMCs). All three extracts exhibited inhibitory activity against both viruses and significantly reduced the expression of inflammatory mediators. Furthermore, the extracts demonstrated complex immunomodulatory effects in bovine PBMCs, with Extract C promoting T helper 1 and T helper 17 responses while suppressing the regulatory T-cell transcription factor forkhead box P3. In conclusion, the results of this study demonstrate that these herbal extracts possess comprehensive therapeutic potential, combining direct antiviral, potent anti-inflammatory, and sophisticated immunomodulatory properties, strongly supporting their potential as promising candidates for the integrated treatment and prevention of neonatal calf diarrhea. |
| **Order of Authors:** | Xi-Rui Xiang |
| | Eun-Seo Lee |
| | Junho Lee |
| | Su Min Min Kyung |
| | Han Sang Yoo |
| **Additional Information:** | |
| **Question** | **Response** |
| **Financial Disclosure**<br><br>Enter a financial disclosure statement that describes the sources of funding for the work included in this submission. Review the submission guidelines for detailed | Yes |

Powered by Editorial Manager® and ProduXion Manager® from Aries Systems Corporation

| | |
|---|---|
| requirements. View published research articles from *PLOS ONE* for specific examples.<br><br>This statement is required for submission and **will appear in the published article** if the submission is accepted. Please make sure it is accurate.<br><br>**Funded studies**<br>Enter a statement with the following details:<br>• Initials of the authors who received each award<br>• Grant numbers awarded to each author<br>• The full name of each funder<br>• URL of each funder website<br>• Did the sponsors or funders play any role in the study design, data collection and analysis, decision to publish, or preparation of the manuscript?<br><br>Did you receive funding for this work? | |
| Please add funding details.<br>as follow-up to "**Financial Disclosure**<br><br>Enter a financial disclosure statement that describes the sources of funding for the work included in this submission. Review the submission guidelines for detailed requirements. View published research articles from *PLOS ONE* for specific examples.<br><br>This statement is required for submission and **will appear in the published article** if the submission is accepted. Please make sure it is accurate. | This study was supported by SMTECH grant number S3346171, the BK21 FOUR program, and the Research Institute for Veterinary Science, Seoul National University, Republic of Korea. |

| | |
|---|---|
| **Funded studies**<br>Enter a statement with the following details:<br>• Initials of the authors who received each award<br>• Grant numbers awarded to each author<br>• The full name of each funder<br>• URL of each funder website<br>• Did the sponsors or funders play any role in the study design, data collection and analysis, decision to publish, or preparation of the manuscript?<br><br>Did you receive funding for this work?" | |
| Please select the country of your main research funder (please select carefully as in some cases this is used in fee calculation).<br>as follow-up to "**Financial Disclosure**<br><br>Enter a financial disclosure statement that describes the sources of funding for the work included in this submission. Review the submission guidelines for detailed requirements. View published research articles from *PLOS ONE* for specific examples.<br><br>This statement is required for submission and **will appear in the published article** if the submission is accepted. Please make sure it is accurate.<br><br>**Funded studies**<br>Enter a statement with the following details:<br>• Initials of the authors who received each award<br>• Grant numbers awarded to each author<br>• The full name of each funder<br>• URL of each funder website<br>• Did the sponsors or funders play any role in the study design, data collection and analysis, decision to publish, or preparation of the manuscript?<br><br>Did you receive funding for this work?" | KOREA, REPUBLIC OF - KR |
| **Competing Interests** | The authors have declared that no competing interests exist. |

| | |
|---|---|
| Use the instructions below to enter a competing interest statement for this submission. On behalf of all authors, disclose any competing interests that could be perceived to bias this work—acknowledging all financial support and any other relevant financial or non-financial competing interests.<br><br>This statement is required for submission and **will appear in the published article** if the submission is accepted. Please make sure it is accurate and that any funding sources listed in your Funding Information later in the submission form are also declared in your Financial Disclosure statement.<br><br>View published research articles from *PLOS ONE* for specific examples.<br><br>**NO authors have competing interests**<br><br>Enter: *The authors have declared that no competing interests exist.*<br><br>**Authors with competing interests**<br><br>Enter competing interest details beginning with this statement:<br><br>*I have read the journal's policy and the authors of this manuscript have the following competing interests: [insert competing interests here]*<br><br>* typeset | |
| **Ethics Statement**<br><br>Enter an ethics statement for this submission. This statement is required if the study involved:<br><br>• Human participants<br>• Human specimens or tissue<br>• Vertebrate animals or cephalopods<br>• Vertebrate embryos or tissues | This study was conducted in an approved facility in strict accordance with all institutional and national guidelines. All animal-related experiments were reviewed and approved by the Institutional Animal Care and Use Committee (IACUC) of Seoul National University, under approval number SNU-240219-4-1. |

- Field research

Write "N/A" if the submission does not require an ethics statement.

General guidance is provided below. Consult the submission guidelines for detailed instructions. **Make sure that all information entered here is included in the Methods section of the manuscript.**

Powered by Editorial Manager® and ProduXion Manager® from Aries Systems Corporation

| | |
|---|---|
| **Format for specific study types**<br><br>**Human Subject Research (involving human participants and/or tissue)**<br>• Give the name of the institutional review board or ethics committee that approved the study<br>• Include the approval number and/or a statement indicating approval of this research<br>• Indicate the form of consent obtained (written/oral) or the reason that consent was not obtained (e.g. the data were analyzed anonymously)<br><br>**Animal Research (involving vertebrate animals, embryos or tissues)**<br>• Provide the name of the Institutional Animal Care and Use Committee (IACUC) or other relevant ethics board that reviewed the study protocol, and indicate whether they approved this research or granted a formal waiver of ethical approval<br>• Include an approval number if one was obtained<br>• If the study involved *non-human primates*, add *additional details* about animal welfare and steps taken to ameliorate suffering<br>• If anesthesia, euthanasia, or any kind of animal sacrifice is part of the study, include briefly which substances and/or methods were applied<br><br>**Field Research**<br><br>Include the following details if this study involves the collection of plant, animal, or other materials from a natural setting:<br>• Field permit number<br>• Name of the institution or relevant body that granted permission | |
| **Data Availability**<br><br>Authors are required to make all data underlying the findings described fully available, without restriction, and from the time of publication. PLOS allows rare exceptions to address legal and ethical concerns. See the PLOS Data Policy and FAQ for detailed information. | Yes - all data are fully available without restriction |

| | |
|---|---|
| A Data Availability Statement describing where the data can be found is required at submission. Your answers to this question constitute the Data Availability Statement and **will be published in the article**, if accepted.<br><br>**Important:** Stating 'data available on request from the author' is not sufficient. If your data are only available upon request, select 'No' for the first question and explain your exceptional situation in the text box.<br><br>Do the authors confirm that all data underlying the findings described in their manuscript are fully available without restriction? | |
| **Describe where the data may be found in full sentences. If you are copying our sample text, replace any instances of XXX with the appropriate details.**<br><br>• If the data are **held or will be held in a public repository**, include URLs, accession numbers or DOIs. If this information will only be available after acceptance, indicate this by ticking the box below. For example: *All XXX files are available from the XXX database (accession number(s) XXX, XXX.*).<br>• If the data are all contained **within the manuscript and/or Supporting Information files**, enter the following: *All relevant data are within the manuscript and its Supporting Information files.*<br>• If neither of these applies but you are able to provide **details of access elsewhere**, with or without limitations, please do so. For example:<br><br>*Data cannot be shared publicly because of [XXX]. Data are available from the XXX Institutional Data Access / Ethics Committee (contact via XXX) for researchers who meet the criteria for access to confidential data.*<br><br>*The data underlying the results presented in the study are available from (include the name of the third party* | All relevant data are within the manuscript and its supporting information files. |

| | |
|---|---|
| *and contact information or URL).*<br>• This text is appropriate if the data are owned by a third party and authors do not have permission to share the data.<br><br>* typeset | |
| Additional data availability information: | Tick here if the URLs/accession numbers/DOIs will be available only after acceptance of the manuscript for publication so that we can ensure their inclusion before publication.; Tick here if your circumstances are not covered by the questions above and you need the journal's help to make your data available. |

Manuscript Click here to access/download;Manuscript;PLoS One text - Xiang.docx

# Antiviral, anti-inflammatory and immunomodulatory effects of herbal extracts of *Boswellia serrata*, *Commiphora myrrha*, *Scutellaria baicalensis*, *Gardenia jasminoides*, *Nypa fruticans* and propolis

**Xi-Rui Xiang, Eun-Seo Lee, Jun Ho Lee, Su Min Kyung, Han Sang Yoo**[*]

Departments of Infectious Disease, College of Veterinary Medicine, Seoul National University, Seoul, Republic of Korea

[*] Corresponding author

Email: yoohs@snu.ac.kr (HY)

# Abstract

Traditional herbal extracts are attracting attention in the context of animal disease control because of their low side effects, diverse bioactive compounds, and low antimicrobial resistance risk. However, the underlying mechanisms remain inadequately understood. To characterize the multifaceted biological activities that underlie their therapeutic potential, this study systematically evaluated the antiviral, anti-inflammatory, and immunomodulatory properties of three herbal extracts *in vitro*. The antiviral activities of the extracts were tested against bovine rotavirus and bovine coronavirus, two major pathogens of neonatal calf diarrhea, and their anti-inflammatory effects were assessed in a lipopolysaccharide-stimulated murine macrophage model (RAW 264.7 cells) by measuring nitric oxide production and the expression of inflammation-related enzymes. Immunomodulatory effects were investigated by analyzing the gene expression of T helper cell-associated cytokines in both RAW 264.7 cells and bovine peripheral blood mononuclear cells (PBMCs). All three extracts exhibited inhibitory activity against both viruses and significantly reduced the expression of inflammatory mediators. Furthermore, the extracts demonstrated complex immunomodulatory effects in bovine PBMCs, with Extract C promoting T helper 1 and T helper 17 responses while suppressing the regulatory T-cell transcription factor forkhead box P3. In conclusion, the results of this study demonstrate that these herbal extracts possess comprehensive therapeutic potential, combining direct antiviral, potent anti-inflammatory, and sophisticated immunomodulatory properties, strongly supporting their potential as promising candidates for the integrated treatment and prevention of neonatal calf diarrhea.



Keywords: Herbal extracts; Bovine rotavirus; Bovine coronavirus; Antiviral activity; Anti-inflammatory effect; Immunomodulation.

# Introduction

Diarrhea is among the leading causes of morbidity and mortality in newborn calves, resulting in substantial economic losses[1, 2] and posing a serious threat to the cattle industry across Asia and worldwide[3-5]. Among the diverse etiologies of the disease, including viruses, bacteria and parasites[6-8], bovine rotavirus (BRV) and bovine coronavirus (BCoV) are the predominant viral pathogens of enteritis[9, 10]. Both viruses severely damage the integrity of the intestinal epithelium, leading to dehydration, electrolyte imbalance and even death[11-13].

At present, clinical treatment strategies for calf diarrhea rely mainly on supportive care[8], and often involve the use of antibiotics to prevent or control secondary bacterial infections[14, 15]. However, the widespread use of antibiotics has exacerbated not only the global crisis of bacterial resistance[16], but also complicated subsequent treatments[5, 17]. Importantly, the damage caused by viral infection extends beyond direct cytopathic injury to the gut epithelium, and frequently involves dysregulated inflammatory responses[18], such as excessive inflammatory reactions and disruption of immune homeostasis[19]. In antiviral host defense, the immune response driven by T cells plays a vital role[20, 21]. CD4+ T helper (Th) cells represent a significant population[20], and include key components such as Th1, Th2, Th17, and regulatory T-cells (Treg) subsets[22]. The balance among these cells is critical, as each produces cytokines and transcription factors that initiate the immune response[23]. Th1 cells promote cellular immunity and antiviral responses through the secretion of cytokines such as interferon-gamma (IFN-γ), interleukin-2 (IL-2), and tumor necrosis factor-alpha (TNF-α), which are regulated by the transcription factor T-box 21 (TBX21, or T-bet)[24], whereas Th2 cells facilitate humoral immunity through cytokines, including IL-4, IL-5, IL-10 and IL-13, under the control of GATA binding protein 3 (GATA3)[25, 26]. Th17 cells, characterized primarily by IL-17 and IL-22, are driven by the transcription factor RAR-related orphan receptor C (RORC), and are involved in mucosal defense and inflammatory pathology[27],

whereas Treg cells produce cytokines such as IL-10 and transforming growth factor-beta (TGF-β) to suppress excessive immune activation, with high expression of cytotoxic T-lymphocyte antigen 4 (CTLA-4) and the transcription factor forkhead box P3 (Foxp3),  to prevent tissue damage[28].

Therefore, an ideal treatment strategy should include direct antiviral activity, effective modulation of the host immune system, and restoration of immune homeostasis. Natural herbs have shown great potential because of their multicomponent and multitarget properties[29, 30]. For example, *Scutellaria baicalensis*[31-33] and *Commiphora myrrha*[34, 35]are known for their potent anti-inflammatory effects. The active component of *Boswellia serrata* has been confirmed to have broad antiviral activity[36-38]. Propolis, on the other hand, has significant immunomodulatory functions[39-41]. On the basis of these theories, three different herbal compound extracts (Extract A, Extract B and Extract C) were scientifically formulated and prepared to identify optimal candidates for the treatment of viral diarrhea in calves.

Leave a space before 'are'

To characterize the multifaceted biological activities that underlie their therapeutic potential, this study systematically evaluated the antiviral, anti-inflammatory, and immunomodulatory properties of the three herbal extracts. The goal of this study was to identify a safe and effective candidate that offers a natural therapeutic option for viral diarrhea in calves and aligns with the "One Health" concept, which emphasizes maintaining ecosystem balance and human well-being by safeguarding animal health.

# Materials and methods

## Herbal materials

Extract A, Extract B, and Extract C used in this study were provided by K Pharms Co., Ltd. (Suwon, Korea). The compositions were as follows: Extract A was composed of *B. serrata, C. myrrha* and propolis,

and Extract B was a single extract of *Nypa fruticans*. Extract C was a compound of *B. serrata, C. myrrha, S. baicalensis, Gardenia jasminoides*, and propolis. All the extracts were dissolved in DMSO to prepare 200 mg/mL stock solutions, which were stored at -20 °C in the dark. Working concentrations of DMSO were freshly prepared immediately before experimentation. The final DMSO concentration did not exceed 0.1% (v/v), and vehicle controls were included accordingly.

## Cells and virus culture

### Cell lines and virus

Madin-Darby Bovine Kidney (MDBK) cells, TF104 cells (a clonal derivative of MA-104 monkey kidney cells), and RAW 264.7 mouse macrophages, all of which were maintained as laboratory stocks, were used in this study. Cells were used at passages 5–10 after thawing. All cells were cultured in Dulbecco's modified Eagle's medium (DMEM; Gibco, USA) supplemented with 10% heat-inactivated fetal bovine serum (FBS; Gibco) and 100 μg/mL streptomycin and 100 U/mL penicillin (Gibco), and incubated at 37 °C and 5% $CO_2$. BCoV (strain number: VR1500040) and BRV (strain number: VR1800005) were kindly provided by Choong Ang Vaccine Laboratories Co., Ltd. (Daejeon, Korea). BCoV was propagated in MDBK cells, and BRV was propagated in TF104 cells. Infectious titers (50% tissue culture infectious dose, $TCID_{50}$) were determined by the Reed–Muench method[42], yielding titers of $1\times10^{5.62}$ $TCID_{50}$/mL for BCoV and $1\times10^{2.86}$ $TCID_{50}$/mL for BRV.

## Isolation and culture of bovine PBMCs

All animal-related procedures were approved by the IACUC of Seoul National University (SNU-240219-4-1). Heparinized jugular venous blood was collected from healthy cattle maintained at the Seoul National

University experimental farm. Bovine peripheral blood mononuclear cells (PBMCs) were isolated by centrifugation at 800 × g for 15 min through Leucosep tubes (Greiner Bio-One, Austria). The PBMC layer was harvested, washed with Dulbecco's phosphate-buffered saline (DPBS; Gibco), and treated with red blood cell lysis buffer (Roche Diagnostics, Germany). The cells were resuspended in RPMI 1640 medium (Gibco), supplemented with 10% heat-inactivated FBS and penicillin–streptomycin (100 U/mL; 100 μg/mL), and then cultivated at 37°C with 5% $CO_2$ until use.

## Cytotoxicity assay

MDBK, TF104, RAW 264.7 cells and bovine PBMCs were seeded in 96-well plates at a density of 1-4×$10^4$ cells/well, respectively. After cell attachment, the culture medium was replaced with fresh medium containing each extract at concentrations of 6.25, 12.5, 25, 50, 100 and 200 μg/mL of each extract. The negative control group (NC) was treated with 0.1% DMSO. For bovine PBMCs, 1 μg/mL lipopolysaccharide (LPS) from *Escherichia coli* (Sigma-Aldrich, USA) was added to the positive control group (PC), which was included as a positive reference for subsequent immunoactivity assays. In accordance with the cell growth characteristics, the viral host cells MDBK and TF104 were treated for 48 hours and 96 hours, respectively, to determine the optimal time points for evaluating the viral cytopathic effect (CPE). The immune cells, RAW 264.7 cells and bovine PBMCs, were treated for 24 hours and 48 hours, respectively, representing the optimal duration for assessing their immunomodulatory responses while maintaining good cell viability.

Adherent cells were processed with MTT reagent (5 mg/mL stock; Thermo Fisher Scientific, USA) according to the manufacturer's instructions. PBMCs received 10 μL of WST-1 (Abcam, UK) per well. The absorbance was measured on a microplate reader at 570 nm for MTT and 450 nm for WST-1.

## RNA extraction and reverse transcription

Total RNA was extracted using TRIzol Reagent (Thermo Fisher Scientific). After chloroform phase separation, the RNA was precipitated with isopropanol, washed with 75% ethanol, and finally resuspended in DEPC-treated water. cDNA was synthesized from total RNA using a High-Capacity cDNA Reverse Transcription Kit (Applied Biosystems, USA) following the manufacturer's protocol and then stored at −20 °C until use.

## Antiviral effect on BCoV and BRV

MDBK and TF104 cells were seeded in 6-well plates and inoculated with either BCoV (MOI = 0.1) or BRV (MOI = 0.01) for 1 hour. A negative control (NC; vehicle-matched 0.1% DMSO) and a virus-only positive control (PC) were included. After adsorption, the inoculum was replaced with medium containing Extracts A–C at 6.25, 12.5 or 25 µg/mL. The cultures were continued for 48 hours in BCoV or 96 hours in BRV until CPE appeared. Afterward, the cells were harvested for RNA extraction and subsequent RT–qPCR analysis.

Relative viral gene expression was quantified by RT-qPCR targeting the BCoV M gene and the BRV VP1 gene on a Rotor-Gene Q (QIAGEN, Germany). Reactions contained 10 µL of EzAmp™ qPCR 2× Master Mix (SYBR Green; ELPIS-BIOTECH, South Korea), 2 µLof cDNA template, and 10 pmol of each primer. Cycling: 95 °C for 3 min; 35 cycles of 95 °C for 15 s and 55-60°C for 55 s. The primers were synthesized by Macrogen Inc. (Seoul, Korea), and the sequences are shown in Table 1. The expression was normalized to that of β-actin and analyzed using the $2^{-\Delta\Delta Ct}$ method[43].

**Table 1. Sequences of primers used for bovine coronavirus and bovine rotavirus viral RNA by RT-qPCR.**

| Targets | Primer names | Sequence 5′–3′ | Accession Number | References |
|---|---|---|---|---|
| Viral RNA | BCoV-F | CTGGAAGTTGGTGGAGTT | KX897162.1 | [43] |
| | BCoV-R | ATTATCGGCCTAACATACATC | | |
| | β-actin-MDBK-F | CTGCGGCATTCACGAAAC | NM_173979.3 | This study |
| | β-actin-MDBK-R | GCAGTGATCTCTTTCTGCATC | | |
| | BRV-F | TTCCGCGCTGCTAACTATGT | PV026141.1 | This study |
| | BRV-R | TGGCAAACTAGCCTCAGTGG | | |
| | β-actin-TF104-F | CGGGACCTGACTGACTACCT | NM_001033084.1 | This study |
| | β-actin-TF104-R | R:CTCCTGCTCGAAGTCCAGG | | |

## Anti-inflammatory effects onRAW264.7 cells

RAW 264.7 cells (1 × $10^4$ cells/well; 96-well plates) were used to evaluate anti-inflammatory activity. In an extract-only check, cells treated with Extracts A–C (6.25–25 μg/mL; vehicle 0.1% DMSO) for 24 hours showed no increase in nitric oxide (NO) by the Griess reagent system (Promega, USA) at 540 nm (data not shown). For the main assay, cells were pretreated with different concentrations (6.25, 12.5 and 25 μg/mL) of the Extracts A–C for 4 hours, and then coincubated with LPS-*E.coli* at 1 μg/mL for 24 hours. A negative control (NC; vehicle only, no LPS) and a positive control (PC; LPS only) were also included. The cell supernatant was collected to quantify production of nitric oxide (NO) by the Griess Reagent System. The cell pellets were harvested for total RNA extraction. The mRNA expression levels of two representative proinflammatory genes, inducible nitric oxide synthase (iNOS) and cyclooxygenase-2 (COX-2), were measured by RT-qPCR with β-actin as the housekeeping gene, according to the method described above. The primers used are listed in Table 2**Error! Reference source not found.**.

**Table 2. Sequences of primer for the detection of inflammation-related genes in RAW 264.7 cells by RT–qPCR.**

| Targets | Primer names | Sequence 5′–3′ | Accession Number | References |
|---|---|---|---|---|

| | | | | |
|---|---|---|---|---|
| Inflammatory cytokines | iNOS-F | GAGCCACAGTCCTCTTTGCT | NM_001429940.1 | This study |
| | iNOS-R | CAACCTTGGTGTTGAAGGCG | | |
| | COX-2-F | TGCCCGACACCTTCAACATT | XM_028775752.1 | This study |
| | COX-2-R | AGAAGCGTTTGCGGTACTC | | |
| | β-actin-RAW-F | GCTCCGGCATGTGCAAAG | NM_007393.5 | This study |
| | β-actin-RAW-R | CCTTCTGACCCATTCCCACC | | |

## Immunomodulatory effects on RAW 264.7 cells and bovine PBMCs

RAW 264.7 macrophages and bovine PBMCs were seeded in 6-well plates at $5 \times 10^5$ and $2–3 \times 10^5$ cells/well, respectively. The cells were treated with different concentrations (6.25, 12.5 and 25 μg/mL) of extracts, LPS-*E. coli* (1 μg/mL, positive control), or 0.1%DMSO (negative control) for 4, 8 and 24 hours in RAW 264.7 cells or 4, 8, 24 and 48 hours in bovine PBMCs. At each time point, total RNA was extracted, and RT–qPCR was performed as previously described, while "relative" fold changes in cytokine expression levels were compared with those in the negative control group by the $2^{-\Delta\Delta Ct}$ method. The targets included immunomodulatory cytokines and transcription factors, as listed in Table 3.

**Table 3. Primer sequences used for the quantification of gene in RAW264.7 cells and bovine peripheral blood mononuclear cells by RT-qPCR.**

| Targets | Primer names | Sequence 5′–3′ | Accession Number | References |
|---|---|---|---|---|
| Genes in RAW264.7 | TNF-α-F | TTCTATGGCCCAGACCCTC | NM_013693.3 | [44] |
| | TNF-α-R | ACTTGGTGGTTTGCTACG | | |
| | IL-5-F | CCATGAGCACAGTGGTGAAAG | NM_010558.1 | This study |
| | IL-5-R | GACAGGAAGCCTCATCGTCTC | | |
| | IL-6-F | TACTCGGCAAACCTAGTGCG | NM_031168.2 | [45] |
| | IL-6-R | GTGTCCCAACATTCATATTGTCAGT | | |
| | IL-10-F | ACCTGGTAGAAGTGATGCCC | NM 010548.2 M | This study |
| | IL-10-R | TGTAGACACCTTGGTCTTGGA | | |

| | | | | |
|---|---|---|---|---|
| | IL-17-F | CTCAGACTACCTCAACCGTTCC | NM_001106897.1 | This study |
| | IL-17-R | GTGCCTCCCAGATCACAGAAG | | |
| | IL-21-F | CAAGCCATCAAACCCTGGAAAC | NM_021782.3 | This study |
| | IL-21-R | TTCTCATACGAATCACAGGAGGG | | |
| | Foxp3-F | CAGACAGCTGGGTTTAAGAT | NM_001199347.2 | This study |
| | Foxp3-R | GATGGATGTCTCCTAACCAC | | |
| | β-actin-F | CATTGCTGACAGGATGCAGAAGG | NM_007393.1 | This study |
| | β-actin-R | TGCTGGAAGGTGGACAGTGAGG | | |
| Genes in PBMCs | IFN-γ-P-F | AATTCCGGTGGATGATCTG | NM_174086.1 | This study |
| | IFN-γ-P-R | GATTCTGACTTCTCTTCCGC | | |
| | T-bet-P-F | ATTACCGGATGTATGTGGAC | NM_001192140 | This study |
| | T-bet-P-R | GCTTTAGTTTCCCGAATGAC | | |
| | IL-4-P-F | GAATTGAGCTTAGGCGTATC | NM_173921.2 | This study |
| | IL-4-P-R | CTTCATTCACAGAACAGGTC | | |
| | GATA3-P-F | CTACCACAAGATGAACGGAC | XM_005214126.5 | This study |
| | GATA3-P-R | AGAGTCGTAGTTGTGGTTTG | | |
| | IL17-P-F | TATGTCACTGCTACTGCTTC | NM_001008412.2 | This study |
| | IL17-P-R | GTTTAGGTTGACCCTCACAT | | |
| | RORC-P-F | CACAGAGACATCACCGAG | NM_001083451 | This study |
| | RORC-P-R | TAGTGGATCCCAGATGACTT | | |
| | CLTA4-P-F | ACTTGGTGGACATCTAGGAC | NM_174297.1 | This study |
| | CLTA4-P-R | GGTCACATTCATCCCTTTAG | | |
| | Foxp3-P-F | TCTATCACTGGTTTACACGC | NM_001045933.1 | This study |
| | Foxp3-P-R | TTGCGGAACTCAAACTCAT | | |
| | β-actin-P-F | GATATTGCTGCGCTCGTGGT | NM_173979 | This study |
| | β-actin-P-R | TACGAGTCCTTCTGGCCCAT | | |

## Statistical analysis

All experiments were repeated a minimum of three times, and the data are presented as the mean ± standard deviation (SD). The information was analyzed using IBM SPSS Statistics 26, and figures were generated

with GraphPad Prism 10.0.0. Comparisons among multiple groups were conducted using one-way analysis of variance (ANOVA). A p value of less than 0.05 was considered to indicate statistical significance (*p < 0.05, **p < 0.01, ***p < 0.001).

# Results

## Cytotoxicity across four cell types

To systematically evaluate cellular safety, four cell types were treated with Extracts A–C, as shown in Fig 1**Error! Reference source not found.**, and their viability is expressed relative to that of the vehicle control (100%). In viral host MDBK cells, only Extract A at 200 μg/mL and Extract C at 100 and 200 μg/mL significantly reduced cell viability to 75.58%, 64.24% and 65.23%, respectively ($p < 0.001$), whereas the other concentrations showed good biological safety. Treatment of TF104 cells for 96 hours resulted in a dose-dependent loss of viability, with 100–200 μg/mL reducing viability to ≤50%. In immune cells, 200 μg/mL Extracts A and C decreased the viability of RAW 264.7 cells to 12.05% and 21.72%, respectively ($p < 0.001$). In contrast, after treatment with bovine PBMCs for 48 hours, the three extracts exhibited no cytotoxicity, and the high-concentration treatment significantly increased cell growth. At 200 μg/mL, Extract C increased the cell viability to 231.97% of that of the control group ($p < 0.001$). Similarly, the PC group showed no significant cytotoxicity to bovine PBMCs. Therefore, based on these findings, noncytotoxic concentrations of 6.25, 12.5, and 25 μg/mL for Extracts A, B, and C, respectively, were selected for all subsequent experiments.

**Fig 1. The extracts have differential cytotoxic effects on different cell types.** MDBK (48 h), TF104 (96 h), RAW264.7 (24 h), and bovine PBMCs (48 h) cells were treated with graded concentrations (6.25–200 μg/mL) of Extract A, B or C. The NC group was treated with vehicle (0.1% DMSO), and the PC group was treated with 1 μg/mL LPS. The data are presented as the mean ± SD (n=4) and are expressed as a percentage of the NC group. Statistical significance relative to the NC group was determined by one-way ANOVA (*p < 0.05, **p < 0.01, ***p < 0.001).

## Antiviral effects on BCoV and BRV

As shown in Fig 2, three concentrations (6.25, 12.5, and 25 μg/mL) of all the extracts significantly inhibited BCoV. Extract A exhibited the strongest suppression at 25 μg/mL (p < 0.001), whereas Extracts B and C showed greater inhibition at 6.25–12.5 μg/mL than at 25 μg/mL. The extracts presented distinct patterns of activity against BRV. The effect of Extract A was strongly dose dependent, with greater inhibition observed at higher doses. At all tested concentrations, Extract B reduced viral replication. However, Extract C showed a biphasic pattern, with an inhibitory effect at the low concentration and a significant increase at the highest concentration of 25 μg/mL (p < 0.001).

**Fig 2. Antiviral effects of the extracts on BCoV and BRV.** MDBK and TF104 cells were infected with BCoV (MOI=0.1) and BRV (MOI=0.01), respectively, and then treated with graded concentrations (6.25–25 μg/mL) of each extract for 48 h or 96 h. The relative mRNA expression of the BCoV M gene and the BRV VP1 gene was quantified by RT-qPCR. NC represents uninfected cells. Data are presented as the mean ± SD (n=3), and statistical significance relative to the virus control (PC; treated with 0.1% DMSO) was determined by one-way ANOVA (**p < 0.01, ***p < 0.001).

## Herbal extracts reduce NO and COX-2 but differentially modulate iNOS

The anti-inflammatory effects of the extracts were evaluated in LPS-stimulated RAW 264.7 cells (Fig 3). At the transcript level, COX-2 mRNA expression was significantly suppressed by all the extracts. The inhibitory effects of Extracts A and C increased with increasing concentration, as Extract B significantly reduced COX-2 expression at all doses ($p < 0.001$). However, with respect to iNOS expression, the results revealed that compared with the positive control, Extract B significantly reduced iNOS expression only at 6.25 μg/mL ($p < 0.01$). Additionally, NO production decreased with all three extracts. It was reduced by Extract C at concentrations ranging from 6.25–25 μg/mL in a dose-dependent manner. At 12.5 and 25 μg/mL, Extract A reduced NO levels ($p < 0.001$), whereas Extract B did so at all the tested doses ($p < 0.05$).

**Fig 3. The extracts suppress inflammatory reactions in LPS-stimulated RAW 264.7 macrophages.** RAW 264.7 cells were treated with 6.25, 12.5 and 25 μg/mL of each extract for 4 h, followed by stimulation with 1 μg/mL LPS-*E. coli* for 24 h. The relative mRNA expression levels of COX-2 and iNOS were determined by RT–qPCR and normalized to that of β-actin. The concentration of nitrite in the cell supernatant was measured using a Griess reagent system. The NC group was left untreated, while the PC group was stimulated with only LPS. The data are presented as the mean ± SD (n=3). Statistical significance relative to the PC group was determined by one-way ANOVA (*$p < 0.05$, **$p < 0.01$, ***$p < 0.001$).

## Extracts induce differential expression of immunomodulatory genes in RAW 264.7 cells

All three extracts induced dynamic and distinct gene expression profiles over a 24-hour period. As shown in Fig 4, 25 μg/mL Extract A caused significant induction of TNF-α expression at 24 hours ($p < 0.001$). This extract also induced peak expression of IL-6, IL-17, and Foxp3 at 8 hours. With respect to Extract B (Fig 5), a concentration of 6.25 μg/mL led to significant expression of Foxp3 at 8 hours ($p < 0.001$). In

contrast, Extract C (Fig 6) at 6.25 μg/mL significantly induced IL-6 expression at 24 hours, whereas it appeared to suppress the expression of Foxp3 throughout the time course. In a separate control experiment (S1 Fig), stimulation with LPS-*E. coli* caused a sustained increase in the expression of most proinflammatory genes, confirming the responsiveness of the assay system.

**Fig 4. Analysis of cytokine gene expression in RAW 264.7 cells stimulated with Extract A.** Asterisks (*p < 0.05, **p < 0.01, ***p < 0.001) indicate that extract-induced gene expression is significantly different from that in the negative control group at the same time points.

**Fig 5. Analysis of cytokine gene expression in RAW 264.7 cells stimulated with Extract B.** Asterisks (*p < 0.05, **p < 0.01, ***p < 0.001) indicate that extract-induced gene expression is significantly different from that in the negative control group at the same time points.

**Fig 6. Analysis of cytokine gene expression in RAW 264.7 cells stimulated with Extract C.** Asterisks (*p < 0.05, **p < 0.01, ***p < 0.001) indicate that extract-induced gene expression is significantly different from that in the negative control group at the same time points.

## Expression of T-cell–associated genes in bovine PBMCs in response to extracts

As shown in Fig 7, all three concentrations of Extract A caused initial upregulation of CTLA4 at 4 hours. The 6.25 μg/mL treatment subsequently increased the TBX21 level at 8 hours ($p < 0.001$), and the 25 μg/mL treatment induced an increase in the IFN-γ level at the final 48-hour time point. Treatment with Extract B at 25 μg/mL resulted in a significant initial upregulation of IFN-γ, TBX21, IL-4, IL-17 and CTLA4 expression (Fig 8; $p < 0.01$). However, all concentrations of Extract B suppressed GATA3 expression throughout the entire time course. In cells treated with Extract C, 25 μg/mL led to significant increases in TBX21 and IL-4 expression at 4 hours, whereas 12.5 μg/mL caused increases in IFN-γ and IL-17 expression at later stages ($p < 0.001$), with recovery of IL-4 and CTLA4 expression observed at 24

hours across all concentrations (Fig 9). After LPS-*E. coli* stimulation, the mRNA expression of IFN-γ, TBX21, IL-17, and CTLA4 increased, peaking at 24 and 48 hours, which established a benchmark for a proinflammatory response (S2 Fig).

**Fig 7. Analysis of cytokine and transcription factor gene expression in bovine peripheral blood mononuclear cells stimulated with Extract A.** Statistical significance was determined relative to that in the negative control group at each time point (*$p < 0.05$, **$p < 0.01$, ***$p < 0.001$).

**Fig 8. Analysis of cytokine and transcription factor gene expression in bovine peripheral blood mononuclear cells stimulated with Extract B.** Statistical significance was determined relative to that in the negative control group at each time point (*$p < 0.05$, **$p < 0.01$, ***$p < 0.001$).

**Fig 9. Analysis of cytokine and transcription factor gene expression in bovine peripheral blood mononuclear cells stimulated with Extract C.** Statistical significance was determined relative to that in the negative control group at each time point (*$p < 0.05$, **$p < 0.01$, ***$p < 0.001$).

# Discussion

Neonatal calf diarrhea remains a persistent challenge to the global cattle industry owing to its high

Kindly delete the space after the bracket

incidence rate and the limitations of available therapies[8, 9, 46] . This multifactorial disease involves both infectious and noninfectious factors, with a high probability of mixed infection by multiple pathogens[47, 48]. As potential and valuable medicinal resources with intrinsic multitarget properties, natural herbs are gaining increasing attention in modern veterinary clinical research. [49, 50]. Although previous studies have reported the efficacy of certain individual herbs[37, 40], the combined antiviral and immunomodulatory effects of combinations of these herbs against BCoV and BRV have not been systematically studied. In this study, three herbal extracts were systematically evaluated, and the results

revealed that all of them exhibited apparent antiviral activity, pronounced anti-inflammatory effects, and the ability to regulate the balance of T-cell subsets. Collectively, these findings support a new comprehensive treatment strategy for calf viral diarrhea through the synergistic effect of multiple targets and provide new ideas for prevention and treatment.

In this study, all three herbal extracts clearly inhibited calf diarrhea viruses (BCoV and BRV) at safe concentrations. Notably, Extracts B and C showed strong antiviral effects even at relatively low concentrations (6.25 and 12.5 μg/mL, respectively). The activity of Extract C is consistent with the reported broad-spectrum antiviral properties of its constituents *B. Serrata* and *S. baicalensis* [36, 51]. However, a complex dose effect was also observed in this study as shown in Fig. 2, at a concentration of 25 μg/mL, Extract C significantly increased BRV replication. Low-dose inhibition and high-dose enhancement indicate complex drug–virus–host interactions. Possible mechanisms include a hormetic response[52], and the noncompetitive interactions of herbal compounds with host entry receptors at defined concentrations[53].

In addition, differences in the inhibitory effects of the extracts against the two viruses were detected: Extract A exhibited stronger inhibitory activity against BCoV than against BRV, whereas Extract C had the opposite effect. This difference, together with compositional differences such as the absence of *S. baicalensis* and *G. jasminoides* between Extracts A and C, supports a composite, multicomponent mechanism. With respect to enveloped BCoV, viral entry steps may occur. For example, *C.myrrha* may mediate the modulation of host membrane mediators in influenza virus[54], and the blockade of β-coronavirus spike glycoprotein interactions as reported for SARS-CoV-2[55]. Although these interactions at the entry level were not directly tested in this study, their relevance to BCoV inhibition warrants further investigation. With respect to nonenveloped BRV, intracellular pathways have been implicated for baicalin from *S. baicalensis* via the p-JNK–PDK1–AKT–SIK2 pathway[56]and for genipin from *G.*

*jasminoides* that exhibits anti-rotavirus activity[57]. Given the complex nature of herb formulations, such interactions may exhibit antagonistic or nonlinear behavior under specific combinations or concentrations. This may explain the anomalous reduction in inhibitory activity observed at high concentrations of Extract C. Similar concentration-dependent antagonism has been observed for other herbal compounds[58], suggesting that rational formulation and dose optimization are essential for future studies.

In the LPS-induced RAW 264.7 macrophage inflammation model, the herbal extracts in this study significantly inhibited inflammation by decreasing the mRNA expression of the key inflammatory mediator COX-2 and the production of NO (Fig 3). In particular, the inhibitory effect of Extract C was dose dependent, which is consistent with the report that *B. serrata*[38] and *C. myrrha*[35] are potent anti-inflammatory herbs. LPS-*E. coli* induces an inflammatory response mainly by activating the NF-κB signaling pathway, which also plays a key regulatory role in intestinal inflammatory conditions such as inflammatory bowel disease[59, 60]. Studies have indicated that compounds such as *S. baicalensis* can exert anti-inflammatory effects through the NF-κB pathway, signaling the downregulation of COX-2 expression[61]. Although NF-κB pathway activation was not directly measured in this study, we hypothesize that the observed inhibition of COX-2 activity may have resulted from upstream interference with this pathway.

Moreover, the extracts also significantly suppressed NO production, suggesting their potential to block the downstream inflammatory responses. However, their effects on iNOS were more complex. Although iNOS is a direct target of NF-κB[62], its transcription is coregulated by multiple proinflammatory cytokines, including TNF-α, IFN-γ, and IL-6[60]. Notably, in unstimulated RAW 264.7 cells, the extracts induced TNF-α and IL-6 (Fig 6) expression. Therefore, predominant constituents may attempt to downregulate iNOS expression via NF-κB, whereas other components may be counteracted by cytokine induction, suggesting that it may have pathway-selective or multidirectional regulatory effects. Although

the direct regulatory mechanism of iNOS by the extract remains to be elucidated, the effective inhibition of COX-2 and NO by the extract supports its potential in inflammation-related disease models[63], which is worthy of further study and optimization in the future.

We used two immune cell models, RAW264.7 macrophages and bovine PBMCs, to evaluate the immunomodulatory effects of the herbal extracts in a comparative manner. As a widely used macrophage line[64], RAW264.7 cells served as a reference for changes in PBMC. They are a major source of a variety of cytokines involved in immune responses, hematopoiesis, inflammation, and other homeostatic processes. After they are stimulated with microbial components such as LPS or endogenous factors such as cytokines, they synthesize and release a variety of mediators. Under basal conditions (without LPS), as shown in Fig 4, Extract A (25 μg/mL) increased TNF-α levels at 24 h and produced 8 h peaks in the expression of IL-6, IL-17, and Foxp3, whereas Extract C, as shown in Fig 6 (6.25 μg/mL), increased the level of IL-6 at 24 h, with Foxp3 expression suppressed throughout. These results suggest that certain herbal components may regulate macrophage phenotypes even in the absence of external stimuli. However, RAW 264.7 cells are not a major source of specific cytokines, such as IL-5 or IL-21. Therefore, only limited changes were observed. In contrast, the PBMC model contains many immune cell types. It showed clearer and more diverse responses[64]. The levels of several cytokines and transcription factors, such as Th1, Th2, Th17, and Treg, changed significantly after extract treatment[24]. These findings suggest that the extracts may have broad immunomodulatory potential in complex immune systems.

As introduced earlier, the dynamic balance among T helper cell subsets is key to immune regulation. As illustrated in Fig 9, 25 μg/mL Extract C induced early Th1/Th2 gene expression. At a concentration of 25 μg/mL, early Th1/Th2 gene expression was induced, as activation of the TBX21 and IL-4 pathways was observed at 4 h, whereas the levels of the Th17 factors IL-17 and RORC predominantly increased at later time points at a lower dose (12.5 μg/mL). The concentration of IFN-γ, a key Th1 cytokine, also

peaked at 48 h at 12.5 μg/mL. In contrast, the regulatory Treg transcription factor Foxp3 was suppressed across all concentrations and times, whereas CTLA4 expression increased and then decreased. These results indicate that Extract C may promote Th1/Th17 responses and the concomitant suppression of Treg signals under certain conditions. These results suggest that the effects of the extracts can modulate T-cell differentiation depending on the treatment conditions. This kind of complex response is often seen in natural products that work through multiple biological targets. For example, propolis has been reported to activate Th1- and Th2-associated cytokines[65]; *B. serrata* has been shown to downregulate TNF-α, IL-6, and IL-17, as well as T-cell activation markers such as CD4 and CD8[66]; and reports concerning *S. baicalensis* have indicated that natural components can alleviate inflammatory responses via the NF-κB and NLRP3 pathways[67]. Collectively, these findings support the potential of compound extracts to coordinate immune responses by modulating representative markers associated with T-cell differentiation.

# Conclusion

In conclusion, the results of this study demonstrate that the three tested herbal extracts have significant and broad therapeutic potential against neonatal calf diarrhea. The extracts exhibited potent direct antiviral activity against both BCoV and BRV, displayed pronounced anti-inflammatory effects through the inhibition of key mediators such as COX-2 and NO in a murine macrophage model, and demonstrated a sophisticated ability to modulate T helper cell subsets in a bovine PBMC model. For instance, Extract C promoted Th1/Th17 responses while suppressing regulatory T-cell markers. The distinct patterns observed across different concentrations, viruses, and immune cell types underscore the multitarget, context-dependent nature of these formulations. Together, these findings support a comprehensive therapeutic strategy that integrates antiviral, anti-inflammatory, and immune-regulating actions and highlight the

potential of compound herbal extracts in the development of effective, multifunctional veterinary treatments.

## Ethics statement

This study was conducted in an approved facility in strict accordance with all institutional and national guidelines. All animal-related experiments were reviewed and approved by the Institutional Animal Care and Use Committee (IACUC) of Seoul National University, under approval number SNU-240219-4-1.

## Competing interests

The authors declare that they have no competing interests.

## Author contributions

XRX and HSY designed and conducted the study. The experiments were performed by XRX, ESL and LJH. Data were analyzed and interpreted by XRX and SMK. The manuscript was drafted by XRX and critically revised to its final version by XRX and HSY.

## Funding

This study was supported by SMTECH grant number S3346171, the BK21 FOUR program, and the Research Institute for Veterinary Science, Seoul National University, Republic of Korea.

## Acknowledgments

We thank K Pharms Co., Ltd. in Suwon, Korea, for providing Extracts A, B, and C used in this study. We also acknowledge the Veterinary Research Institute of Seoul National University for providing access to the laboratory facilities used in this study.

# References


1. Cho Y-i, Yoon K-J. An overview of calf diarrhea - infectious etiology, diagnosis, and intervention. Journal of Veterinary Science. 2014;15(1):1-17. doi: 10.4142/jvs.2014.15.1.1.
2. Uetake K. Newborn calf welfare: A review focusing on mortality rates. Animal Science Journal. 2013;84(2):101-5. doi: 10.1111/asj.12019.
3. Zhu Q, Qi S, Guo D, Li C, Su M, Wang J, et al. A survey of fecal virome and bacterial community of the diarrhea-affected cattle in Northeast China reveals novel disease-associated ecological risk factors. Msystems. 2024;9(1):e00842-23. doi: 10.1128/msystems.00842-23.
4. Heinrichs A, Heinrichs B. A prospective study of calf factors affecting first-lactation and lifetime milk production and age of cows when removed from the herd. Journal of Dairy Science. 2011;94(1):336-41. doi: 10.3168/jds.2010-3170.
5. Ali A, Liaqat S, Tariq H, Abbas S, Arshad M, Li W-J, et al. Neonatal calf diarrhea: A potent reservoir of multi-drug resistant bacteria, environmental contamination and public health hazard in Pakistan. Science of the Total Environment. 2021;799:149450. doi: 10.1016/j.scitotenv.2021.149450.
6. Muktar Y, Mamo G, Tesfaye B, Belina D. A review on major bacterial causes of calf diarrhea and its diagnostic method. Journal of Veterinary Medicine and Animal Health. 2015;7(5):173-85. doi: 10.5897/JVMAH2014.0351.
7. Wu Q, Li J, Wang W, Zhou J, Wang D, Fan B, et al. Next-generation sequencing reveals four novel viruses associated with calf diarrhea. Viruses. 2021;13(10):1907. doi: 10.3390/v13101907.
8. Constable PD. Treatment of calf diarrhea: antimicrobial and ancillary treatments. Veterinary Clinics of North America: Food Animal Practice. 2009;25(1):101-20. doi: 10.1016/j.cvfa.2008.10.012.
9. Lee S-H, Kim H-Y, Choi EW, Kim D. Causative agents and epidemiology of diarrhea in Korean native calves. Journal of Veterinary Science. 2019;20(6):e64. doi: 10.4142/jvs.2019.20.e64.
10. Li S, Huang J, Cai X, Mao L, Xie L, Wang F, et al. Prevalence and evolutionary characteristics of bovine coronavirus in China. Veterinary Sciences. 2024;11(6):230. doi: 10.3390/vetsci11060230.
11. Parkhe P, Verma S. Evolution, interspecies transmission, and zoonotic significance of animal coronaviruses. Frontiers in Veterinary Science. 2021;8:719834. doi: 10.3389/fvets.2021.719834.
12. Kostanić V, Kunić V, Prišlin Šimac M, Lolić M, Sukalić T, Brnić D. Comparative Insights into Acute Gastroenteritis in Cattle Caused by Bovine Rotavirus A and Bovine Coronavirus. Veterinary Sciences. 2024;11(12):671. doi: 10.3390/vetsci11120671.
13. Dhama K, Chauhan R, Mahendran M, Malik S. Rotavirus diarrhea in bovines and other domestic animals. Veterinary Research Communications. 2009;33(1):1-23. doi: 10.1007/s11259-008-9070-x.
14. Bernal-Córdoba C, Branco-Lopes R, Latorre-Segura L, de Barros-Abreu M, Fausak E, Silva-del-Río N. Use of antimicrobials in the treatment of calf diarrhea: a systematic review. Animal Health Research Reviews. 2022;23(2):101-12. doi: 10.1017/S1466252322000032.
15. Eibl C, Bexiga R, Viora L, Guyot H, Félix J, Wilms J, et al. The antibiotic treatment of calf diarrhea in four European countries: a survey. Antibiotics. 2021;10(8):910. doi: 10.3390/antibiotics10080910.

16.Santos FdR, De Paula M, Lezier D, Silva Jd, Santos G, Bittar CMM. Essential oils for dairy calves: effects on performance, scours, rumen fermentation and intestinal fauna. Animal. 2015;9(6):958-65. doi: 10.1017/S175173111500018X.
17.Afema JA, Davis MA, Sischo WM. Antimicrobial use policy change in pre-weaned dairy calves and its impact on antimicrobial resistance in commensal Escherichia coli: a cross sectional and ecological study. BMC Microbiology. 2019;19(1):217. doi: 10.1186/s12866-019-1576-6.
18.Foster DM, Smith GW. Pathophysiology of Diarrhea in Calves. Veterinary Clinics of North America Food Animal Practice. 2009;25(1):13-36. doi: 10.1016/j.cvfa.2008.10.013.
19.Fu ZL, Yang Y, Ma L, Malmuthuge N, Guan LL, Bu DP. Dynamics of oxidative stress and immune responses in neonatal calves during diarrhea. Journal of Dairy Science. 2024;107(2):1286-98. doi: 10.3168/jds.2023-23630.
20.Kapil S, Goyal SM, Trent AM. Cellular immune status of coronavirus-infected neonatal calves. Comparative Immunology, Microbiology and Infectious Diseases. 1994;17(2):133-8. doi: 10.1016/0147-9571(94)90038-8.
21.Vlasova AN, Saif LJ. Bovine immunology: Implications for dairy cattle. Frontiers in Immunology. 2021;12:643206. doi: 10.3389/fimmu.2021.643206.
22.Peck A, Mellins ED. Plasticity of T-cell phenotype and function: the T helper type 17 example. Immunology. 2010;129(2). doi: 10.1111/j.1365-2567.2009.03189.x.
23.Cavaillon J. Cytokines and macrophages. Biomedicine & Pharmacotherapy. 1994;48(10):445-53. doi: 10.1016/0753-3322(94)90005-1.
24.Leite F, Kuckleburg C, Atapattu D, Schultz R, Czuprynski C. BHV-1 infection and inflammatory cytokines amplify the interaction of Mannheimia haemolytica leukotoxin with bovine peripheral blood mononuclear cells in vitro. Veterinary Immunology and Immunopathology. 2004;99(3-4):193-202. doi: 10.1016/j.vetimm.2004.02.004.
25.Mosmann TR, Cherwinski H, Bond MW, Giedlin MA, Coffman RL. Two types of murine helper T cell clone. I. Definition according to profiles of lymphokine activities and secreted proteins. Journal of Immunology (Baltimore, MD: 1950). 1986;136(7):2348-57.
26.Brown W, Rice-Ficht AC, Estes DM. Bovine type 1 and type 2 responses. Veterinary Immunology and Immunopathology. 1998;63(1-2):45-55. doi: 10.1016/S0165-2427(98)00081-6.
27.Cunha P, Vern YL, Gitton C, Germon P, Foucras G, Rainard P. Expansion, isolation and first characterization of bovine Th17 lymphocytes. Scientific Reports. 2019;9(1):16115. doi: 10.1038/s41598-019-52562-2.
28.Paust S, Cantor H. Regulatory T cells and autoimmune disease. Immunological Reviews. 2005;204(1):195-207. doi: 10.1111/j.0105-2896.2005.00247.x.
29.Parham S, Kharazi AZ, Bakhsheshi-Rad HR, Nur H, Ismail AF, Sharif S, et al. Antioxidant, antimicrobial and antiviral properties of herbal materials. Antioxidants. 2020;9(12):1309. doi: 10.3390/antiox9121309.
30.Yang C, Li D, Ko C-N, Wang K, Wang H. Active ingredients of traditional Chinese medicine for enhancing the effect of tumor immunotherapy. Frontiers in Immunology. 2023;14:1133050. doi: 10.3389/fimmu.2023.1133050.
31.Liao H, Ye J, Gao L, Liu Y. The main bioactive compounds of Scutellaria baicalensis Georgi. for alleviation of inflammatory cytokines: A comprehensive review. Biomedicine & Pharmacotherapy. 2021;133:110917. doi: 10.1016/j.biopha.2020.110917.
32.Olagaray K, Brouk M, Mamedova L, Sivinski S, Liu H, Robert F, et al. Dietary supplementation of Scutellaria baicalensis extract during early lactation decreases milk somatic cells and increases whole lactation milk yield in dairy cattle. PLoS One. 2019;14(1):e0210744. doi: 10.1371/journal.pone.0210744.
33.Yin B, Li W, Qin H, Yun J, Sun X. The use of Chinese skullcap (Scutellaria baicalensis) and its extracts for sustainable animal production. Animals. 2021;11(4):1039. doi: 10.3390/ani11041039.

34.Batiha GE-S, Wasef L, Teibo JO, Shaheen HM, Zakariya AM, Akinfe OA, et al. Commiphora myrrh: a phytochemical and pharmacological update. Naunyn-Schmiedeberg's Archives of Pharmacology. 2023;396(3):405-20. doi: 10.1007/s00210-022-02325-0.
35.Su S, Wang T, Duan J-A, Zhou W, Hua Y-Q, Tang Y-P, et al. Anti-inflammatory and analgesic activity of different extracts of Commiphora myrrha. Journal of Ethnopharmacology. 2011;134(2):251-8. doi: 10.1016/j.jep.2010.12.003.
36.Gomaa AA, Mohamed HS, Abd-Ellatief RB, Gomaa MA. Boswellic acids/Boswellia serrata extract as a potential COVID-19 therapeutic agent in the elderly. Inflammopharmacology. 2021;29(4):1033-48. doi: 10.1007/s10787-021-00841-8.
37.Hussain H, Wang D, El-Seedi HR, Rashan L, Ahmed I, Abbas M, et al. Therapeutic potential of boswellic acids: an update patent review (2016–2023). Expert Opinion on Therapeutic Patents. 2024;34(8):723-32. doi: 10.1080/13543776.2024.2369626.
38.Siddiqui MZ. Boswellia serrata, a potential antiinflammatory agent: an overview. Indian Journal of Pharmaceutical Sciences. 2011;73(3):255. doi: 10.4103/0250-474X.93507.
39.Araujo MA, Libério SA, Guerra RN, Ribeiro MNS, Nascimento FR. Mechanisms of action underlying the anti-inflammatory and immunomodulatory effects of propolis: a brief review. Revista Brasileira de Farmacognosia. 2012;22:208-19. doi: 10.1590/S0102-695X2011005000167.
40.Yosri N, Abd El-Wahed AA, Ghonaim R, Khattab OM, Sabry A, Ibrahim MA, et al. Anti-viral and immunomodulatory properties of propolis: Chemical diversity, pharmacological properties, preclinical and clinical applications, and in silico potential against SARS-CoV-2. Foods. 2021;10(8):1776. doi: 10.3390/foods10081776.
41.Zhu H, Li C, Jia L, Qiao J, El-Seedi HR, Zhang Y, et al. Supercritical CO2 extracts of propolis inhibits tumor proliferation and Enhances the immunomodulatory activity via activating the TLR4-MAPK/NF-κB signaling pathway. Food Research International. 2024;196:115137. doi: 10.1016/j.foodres.2024.115137.
42.Lj R. A simple method of estimating fifty per cent endpoints. American Journal of Epidemiology. 1938;27:493-5.
43.Decaro N, Elia G, Campolo M, Desario C, Mari V, Radogna A, et al. Detection of bovine coronavirus using a TaqMan-based real-time RT-PCR assay. Journal of Virological Methods. 2008;151(2):167-71. doi: 10.1016/j.jviromet.2008.05.016.
44.Zhao G, Wu H, Jiang K, Rui G, Zhu Z, Qiu C, et al. IFN-τ inhibits S. aureus-induced inflammation by suppressing the activation of NF-κB and MAPKs in RAW 264.7 cells and mice with pneumonia. International Immunopharmacology. 2016;35:332-40. doi: 10.1016/j.intimp.2016.02.016.
45.Wu F, Zhou C, Zhou D, Ou S, Liu Z, Huang H. Immune-enhancing activities of chondroitin sulfate in murine macrophage RAW 264.7 cells. Carbohydrate Polymers. 2018;198:611-9. doi: 10.1016/j.carbpol.2018.06.071.
46.Meale SJ, Chaucheyras-Durand F, Berends H, Guan L, Steele MA. From pre- to postweaning: Transformation of the young calf's gastrointestinal tract. Journal of Dairy Science. 2017;100(7):5984-95. doi: 10.3168/jds.2016-12474.
47.García A, Ruiz-Santa-Quiteria JA, Orden JA, Cid D, Fuente RDL. Rotavirus and concurrent infections with other enteropathogens in neonatal diarrheic dairy calves in Spain. Comparative Immunology Microbiology & Infectious Diseases. 2000;23(3):175-83. doi: 10.1016/S0147-9571(99)00071-5.
48.Meganck V, Hoflack G, Piepers S, Opsomer G. Evaluation of a protocol to reduce the incidence of neonatal calf diarrhoea on dairy herds. Preventive Veterinary Medicine. 2015;118(1):64-70. doi: 10.1016/j.prevetmed.2014.11.007.
49.Li S, Sui M, Wu BY, Langqun. Effects of Chinese herbal plant extracts on diarrhea rate, intestinal morphology, nutrient digestibility, and immunity of weaned piglets. Canadian Journal of Animal Science. 2023;103(4):388-93. doi: 10.1139/cjas-2023-0008.

50.Ma Y-L, Wu Z-M, Liu X, Lan J-E, Zai W-J, Jin X, et al. Antidiarrheal activity of the extracts of Valeriana jatamansi Jones on castor oil-induced diarrhea mouse by regulating multiple signal pathways. Journal of Ethnopharmacology. 2022;298:115560. doi: 10.1016/j.jep.2022.115560.
51.von Rhein C, Weidner T, Henß L, Martin J, Weber C, Sliva K, et al. Curcumin and Boswellia serrata gum resin extract inhibit chikungunya and vesicular stomatitis virus infections in vitro. Antiviral Research. 2016;125:51-7. doi: 10.1016/j.antiviral.2015.11.007.
52.Wang D, Calabrese EJ, Lian B, Lin Z, Calabrese V. Hormesis as a mechanistic approach to understanding herbal treatments in traditional Chinese medicine. Pharmacology & Therapeutics. 2018;184:42-50. doi: 10.1016/j.pharmthera.2017.10.013.
53.Wen Y-Q, Wang Y-Z, Zhao C-X, Zhao B-Y, Wang J-G. Baicalin: an active natural product with potential medicinal values. Journal of Asian Natural Products Research. 2025:1-25. doi: 10.1080/10286020.2025.2482054.
54.Madia VN, De Angelis M, De Vita D, Messore A, De Leo A, Ialongo D, et al. Investigation of Commiphora myrrha (Nees) Engl. oil and its main components for antiviral activity. Pharmaceuticals. 2021;14(3):243. doi: 10.3390/ph14030243.
55.Kulkarni SA, Nagarajan SK, Ramesh V, Palaniyandi V, Selvam SP, Madhavan T. Computational evaluation of major components from plant essential oils as potent inhibitors of SARS-CoV-2 spike protein. Journal of Molecular Structure. 2020;1221:128823. doi: 10.1016/j.molstruc.2020.128823.
56.Song L, Zhong P, Zhu X, Zhou R, Gao M, Lan Q, et al. The anti-rotavirus effect of baicalin via the gluconeogenesis-related p-JNK–PDK1–AKT–SIK2 signaling pathway. European Journal of Pharmacology. 2021;897:173927. doi: 10.1016/j.ejphar.2021.173927.
57.Kim J-H, Kim K, Kim W. Genipin inhibits rotavirus-induced diarrhea by suppressing viral replication and regulating inflammatory responses. Scientific Reports. 2020;10(1):15836. doi: 10.1038/s41598-020-72968-7.
58.Déciga-Campos M, Beltrán-Villalobos KL, Aguilar-Mariscal H, González-Trujano ME, Ángeles-López GE, Ventura-Martínez R. Synergistic herb-herb interaction of the antinociceptive and anti-inflammatory effects of Syzygium aromaticum and Rosmarinus officinalis combination. Evidence-based Complementary and Alternative Medicine. 2021;2021(1):8916618. doi: 10.1155/2021/8916618.
59.Piechota-Polanczyk A, Fichna J. Review article: the role of oxidative stress in pathogenesis and treatment of inflammatory bowel diseases. Naunyn-Schmiedeberg's Archives of Pharmacology. 2014;387(7):605-20. doi: 10.1007/s00210-014-0985-1.
60.Coutinho LL, Femino EL, Gonzalez AL, Moffat RL, Heinz WF, Cheng RY, et al. NOS2 and COX-2 Co-expression promotes cancer progression: a potential target for developing agents to prevent or treat highly aggressive breast cancer. International Journal of Molecular Sciences. 2024;25(11):6103. doi: 10.3390/ijms25116103.
61.Laurindo LF, Santos ARdOd, Carvalho ACAd, Bechara MD, Guiguer EL, Goulart RdA, et al. Phytochemicals and regulation of NF-kB in inflammatory bowel diseases: an overview of in vitro and in vivo effects. Metabolites. 2023;13(1):96. doi: 10.3390/metabo13010096.
62.Farlik M, Reutterer B, Schindler C, Greten F, Vogl C, Müller M, et al. Nonconventional initiation complex assembly by STAT and NF-κB transcription factors regulates nitric oxide synthase expression. Immunity. 2010;33(1):25-34. doi: 10.1016/j.immuni.2010.07.001.
63.Facchin BM, Dos Reis GO, Vieira GN, Mohr ETB, da Rosa JS, Kretzer IF, et al. Inflammatory biomarkers on an LPS-induced RAW 264.7 cell model: A systematic review and meta-analysis. Inflammation Research. 2022;71(7):741-58. doi: 10.1007/s00011-022-01584-0.
64.Elisia I, Pae HB, Lam V, Cederberg R, Hofs E, Krystal G. Comparison of RAW264. 7, human whole blood and PBMC assays to screen for immunomodulators. Journal of Immunological Methods. 2018;452:26-31. doi: 10.1016/j.jim.2017.10.004.

65.Ma X, Guo Z, Li Y, Yang K, Li X, Liu Y, et al. Phytochemical constituents of propolis flavonoid, immunological enhancement, and anti-porcine parvovirus activities isolated from propolis. Frontiers in Veterinary Science. 2022;9:857183. doi: 10.3389/fvets.2022.857183.
66.D'Amico R, Fusco R, Cordaro M, Interdonato L, Crupi R, Gugliandolo E, et al. Modulation of NRF-2 pathway contributes to the therapeutic effects of Boswellia serrata gum resin extract in a model of experimental autoimmune myocarditis. Antioxidants. 2022;11(11):2129. doi: 10.3390/antiox11112129.
67.Zhu Y, Zhu H, Wang Z, Gao F, Wang J, Zhang W. Wogonoside alleviates inflammation induced by traumatic spinal cord injury by suppressing NF-κB and NLRP3 inflammasome activation. Experimental and Therapeutic Medicine. 2017;14(4):3304-8. doi: 10.3892/ETM.2017.4904.

# Supporting information

**S1 Fig. Analysis of cytokine gene expression in RAW 264.7 cells stimulated with a positive control.** The positive control contained 1 μg/mL lipopolysaccharide derived from *Escherichia coli.* Asterisks (**p < 0.01, ***p < 0.001) indicate significant differences compared with the negative control group at the same time points.

**S2 Fig. Analysis of immunomodulatory gene expression in bovine peripheral blood mononuclear cells stimulated with a positive control.** The positive control contained 1 μg/mL lipopolysaccharide derived from *Escherichia coli.* Asterisks (**p < 0.01, ***p < 0.001) indicate significant differences compared with the negative control group at the same time points.

Figure

Click here to access/download;Figure;Fig 1.tif

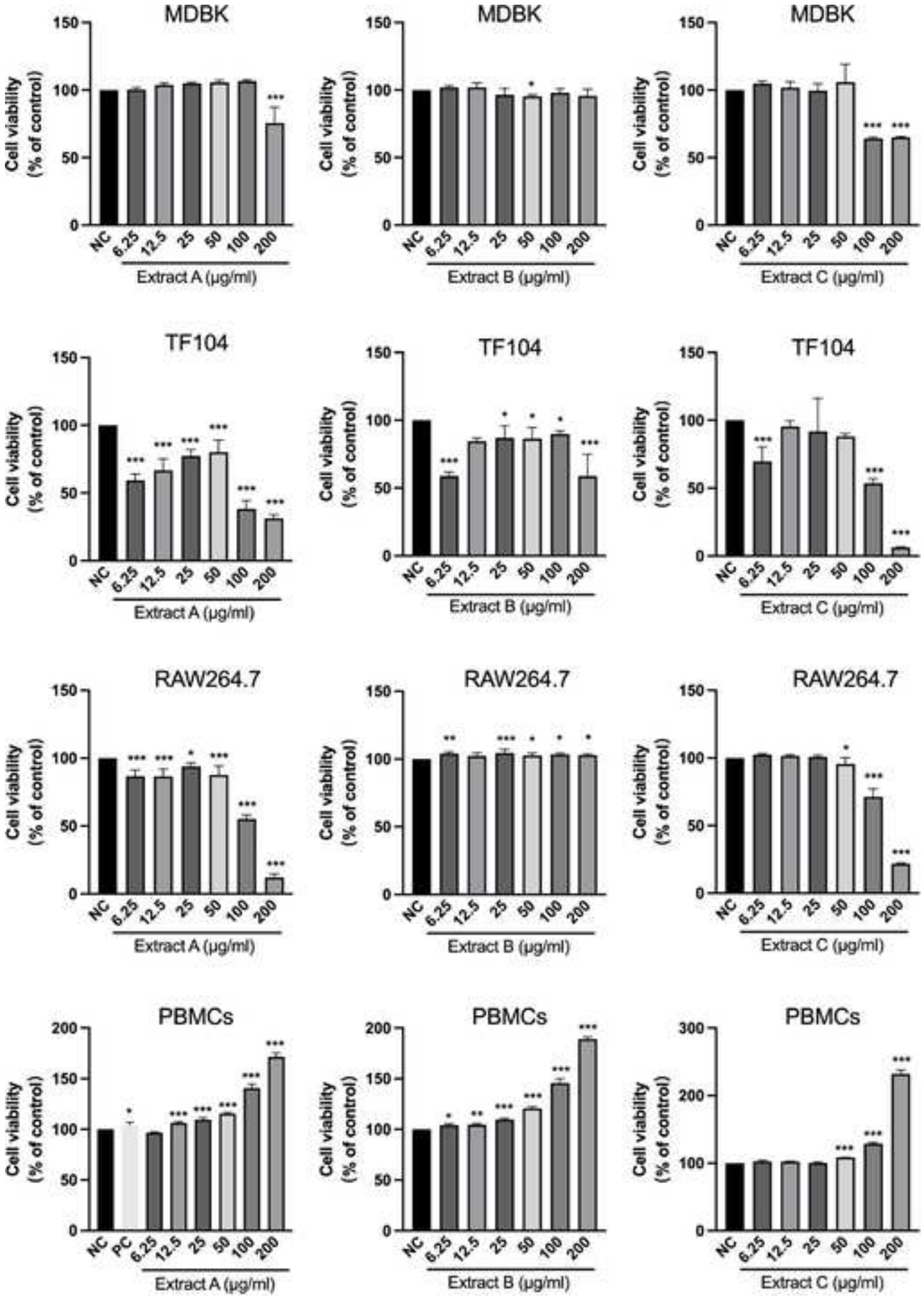

Figure
Click here to access/download;Figure;Fig 2.tif

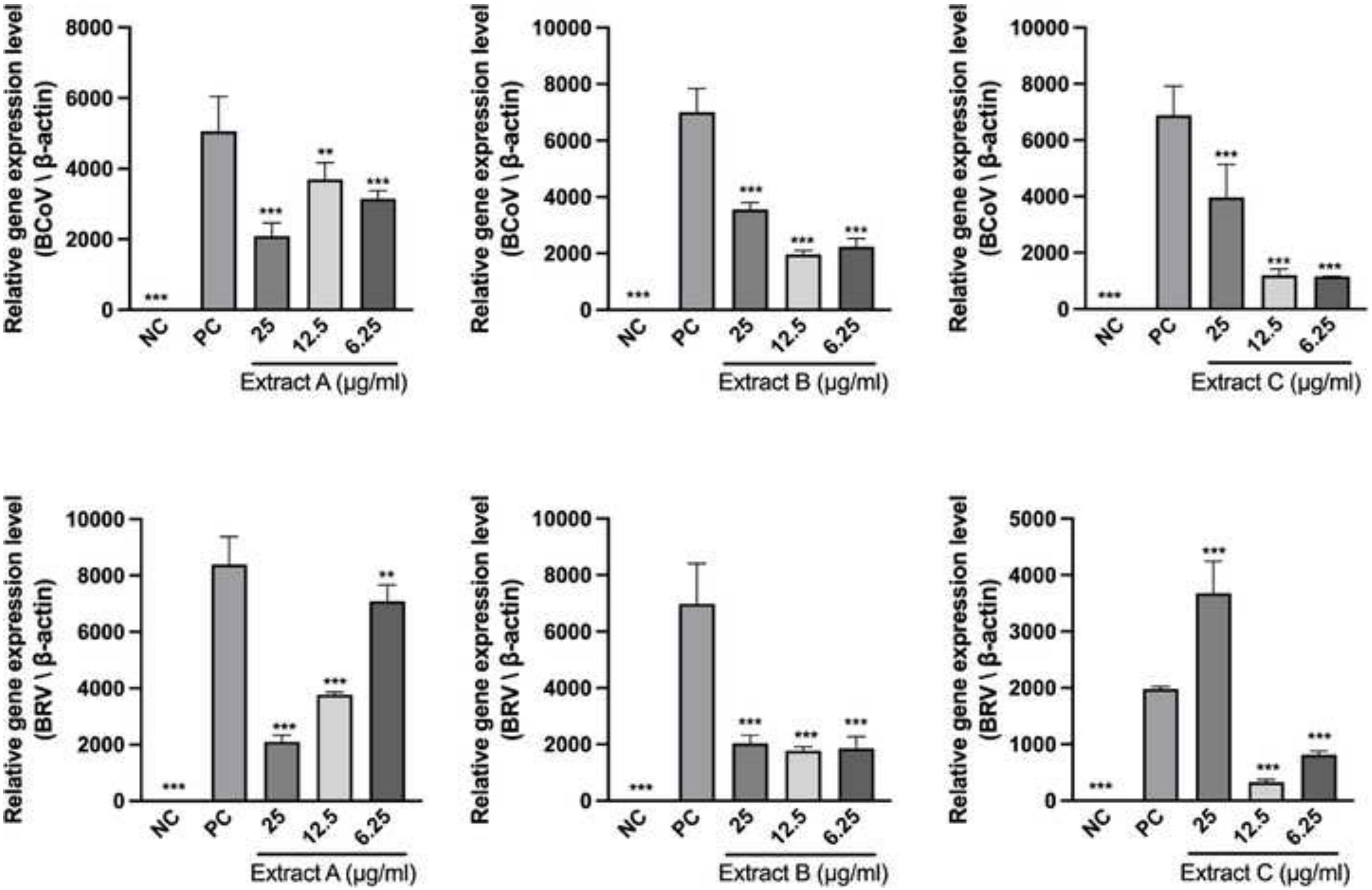

Figure

Click here to access/download;Figure;Fig 3.tif

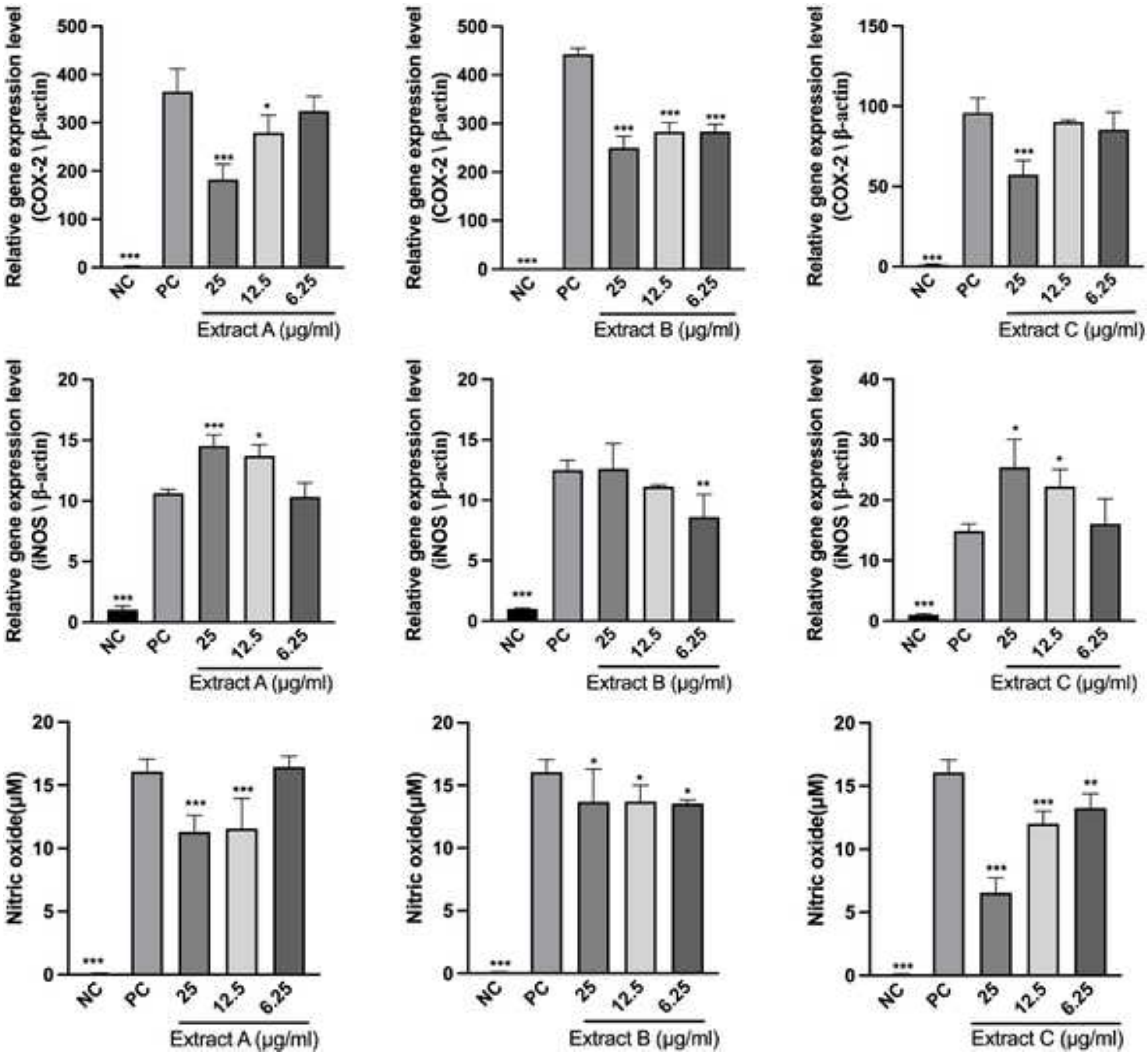

 Click here to access/download;Figure;Fig 4.tif

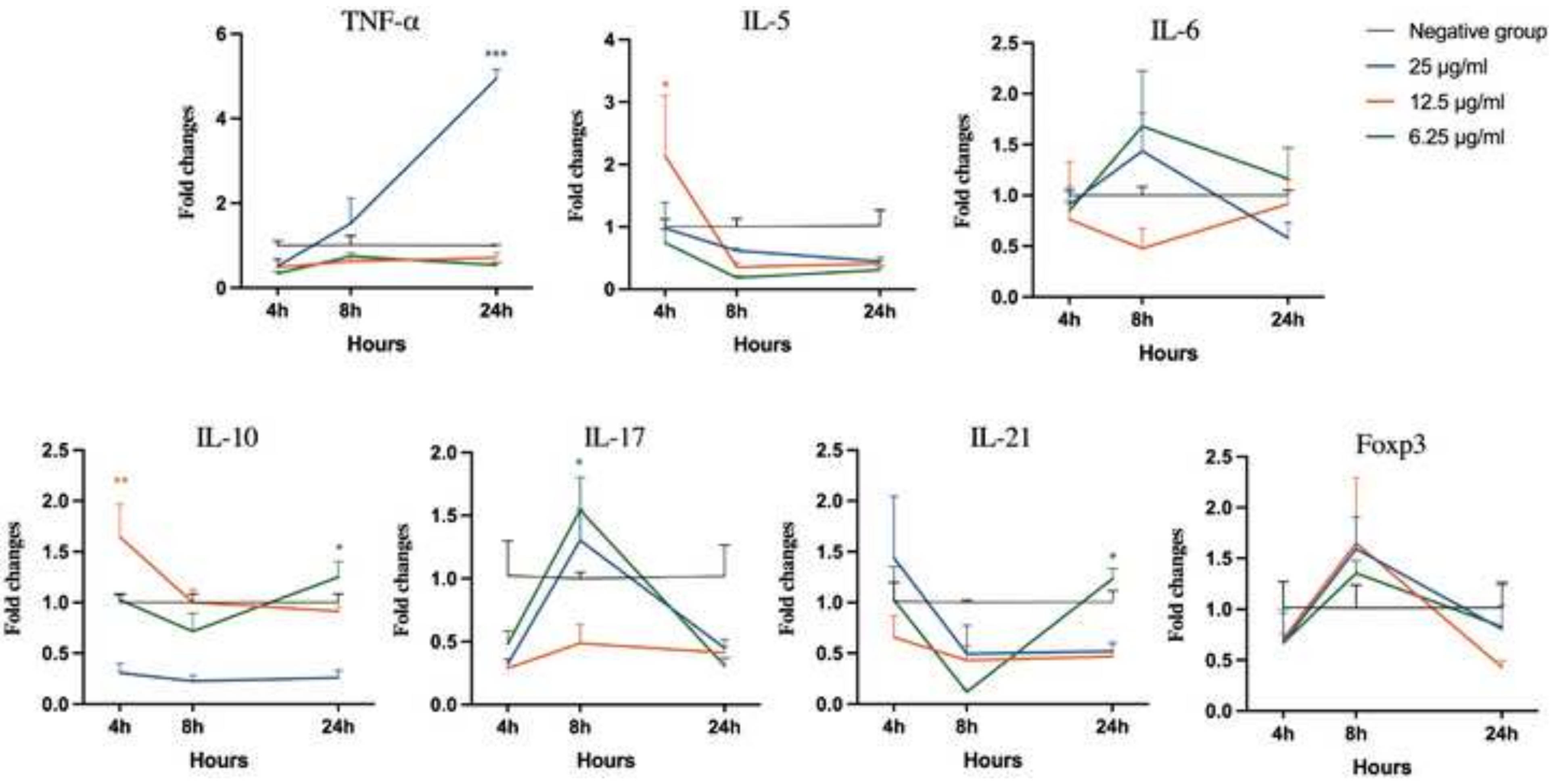

Figure

Click here to access/download;Figure;Fig 5.tif

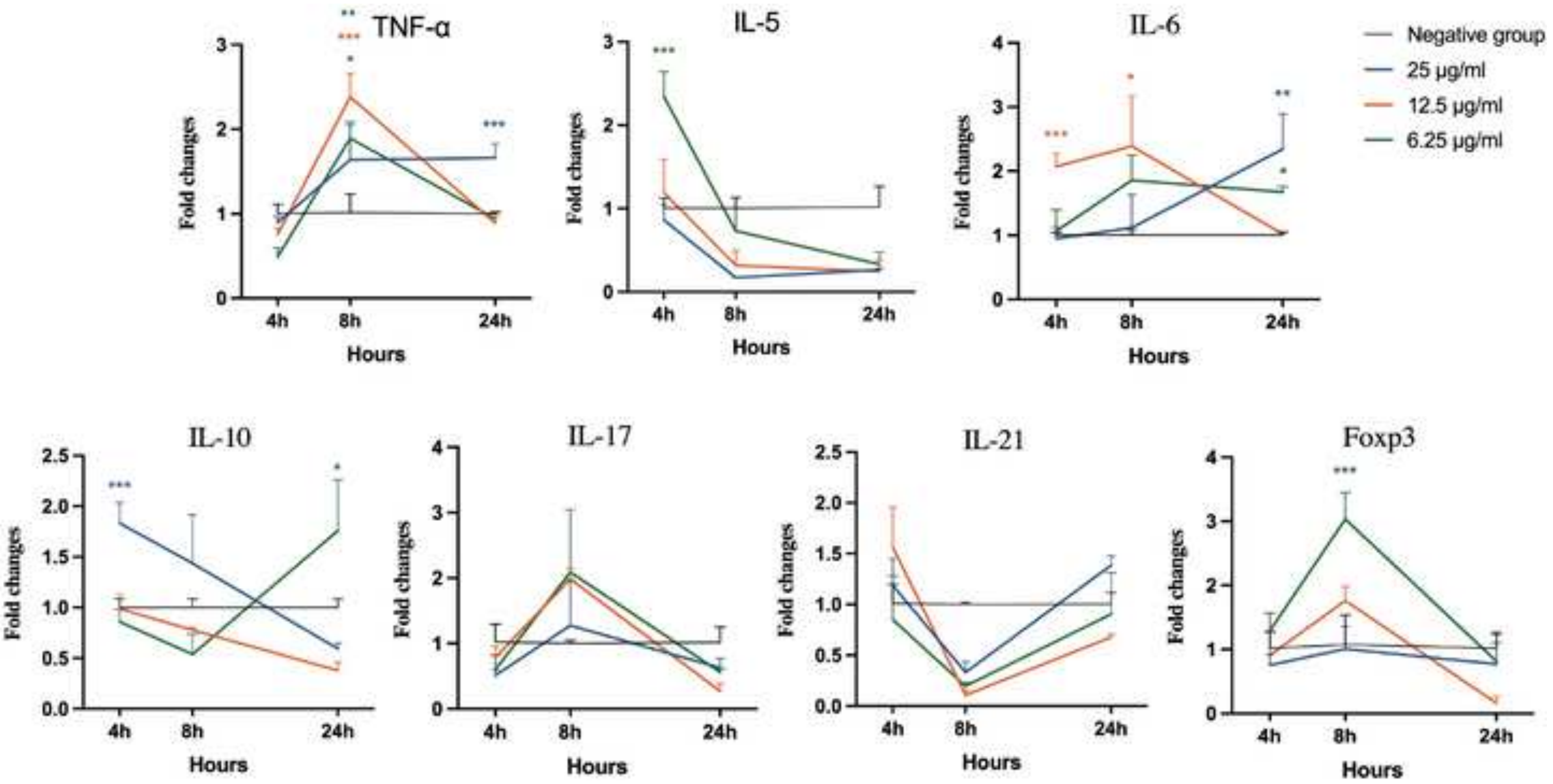

 Click here to access/download;Figure;Fig 6.tif

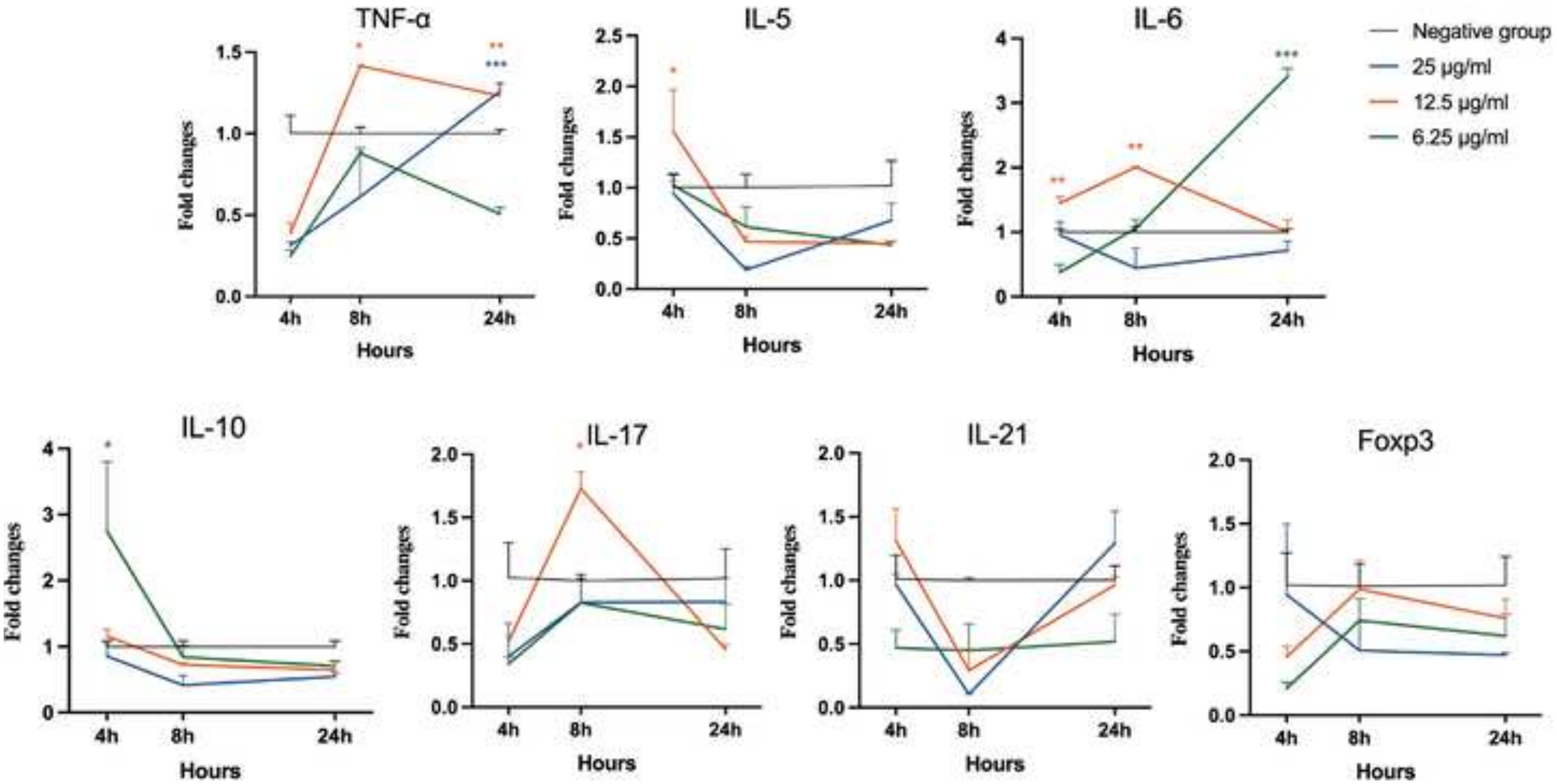

 Click here to access/download;Figure;Fig 7.tif

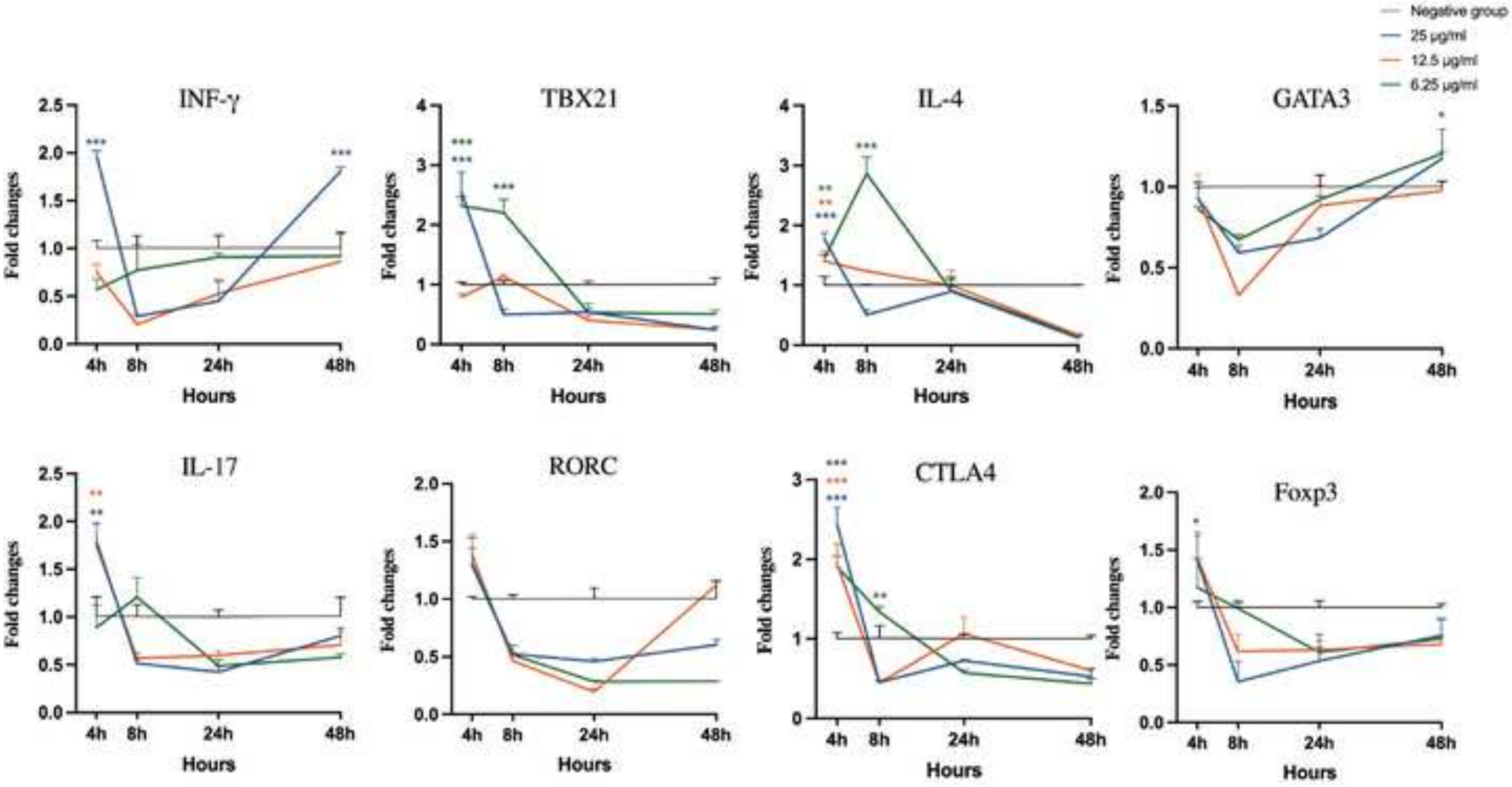

Click here to access/download;Figure;Fig 8.tif

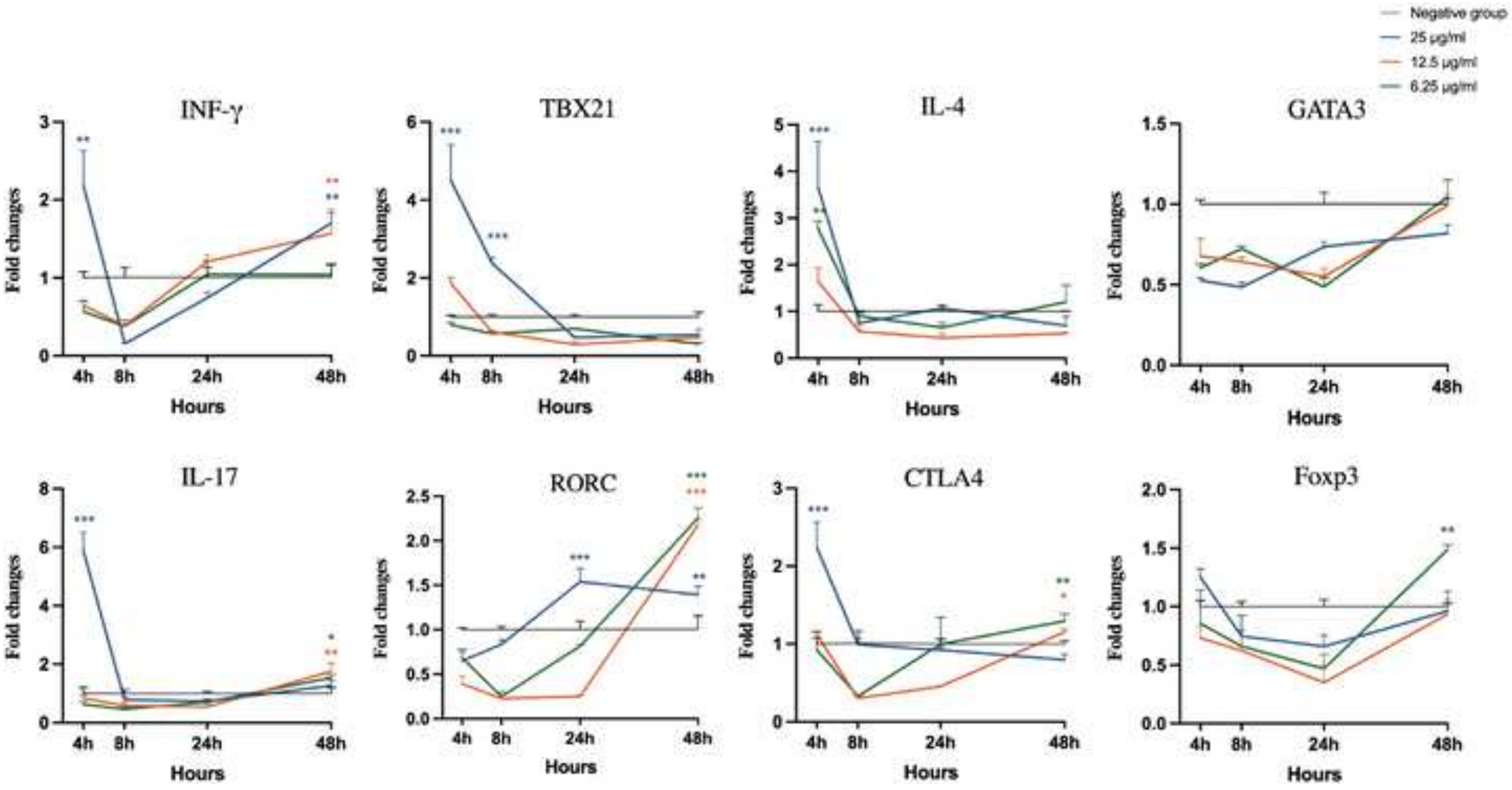

Figure

Click here to access/download;Figure;Fig 9.tif

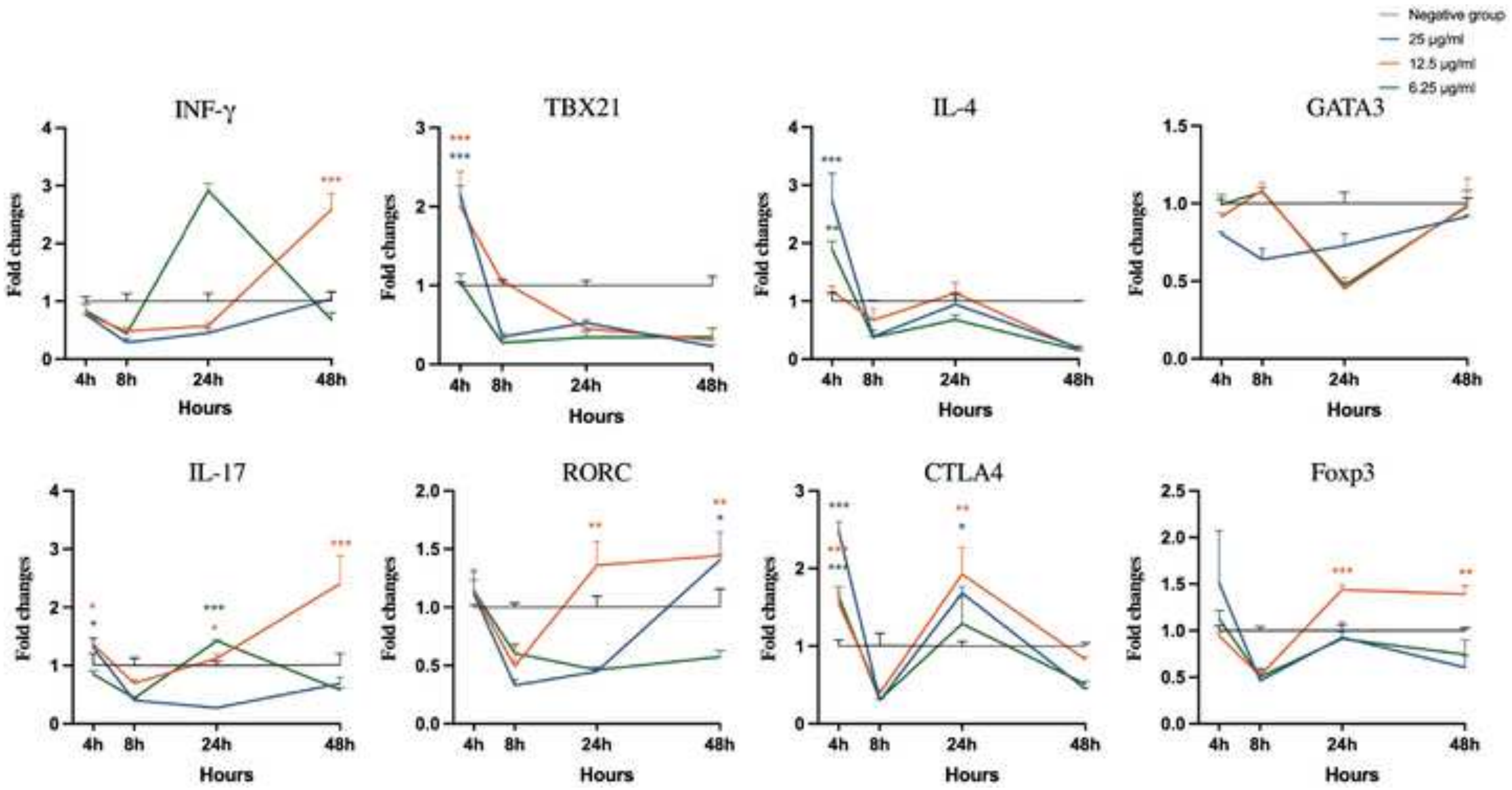

Click here to access/download
**Supporting Information**
S1 Fig.tif

Click here to access/download
**Supporting Information**
S2 Fig.tif
