# Supplementary material for: Antiviral and anti-inflammatory evaluation of herbal extracts: Implications for the management of calf diarrheal diseases
Source: PLoS One. 2026 Feb 6;21(2):e0342013. doi: 10.1371/journal.pone.0342013 (PMC12880661; doi:10.1371/journal.pone.0342013)
Supplement: Attachment — Submitted filename: Response Letter to Reviewers.pdf [file pone.0342013.s006.pdf]

Response Letter to Reviewers' Comments

Dear Editor and Reviewers,

Thank you very much for your continued guidance. We have carefully addressed the additional comments from the latest review round.

Specifically, we have updated the manuscript title to highlight the clinical application, clarified the manufacturer details in the Methods section, and refined the Discussion to provide better mechanistic insights.

Our detailed responses follow below.

● Black text	indicates document navigational headings and identifiers.
● Blue text	represents the original comments from the editor and reviewers.
● Purple text	indicates our direct responses to the editor's and reviewers' comments.

Additionally, we have prepared a comprehensive table of contents to aid navigation and enable quick access to specific sections.

Table of Contents

REVIEWER #1 COMMENTS AND RESPONSE: 2
REVIEWER #3 COMMENTS AND RESPONSE: 3

Reviewer #1 Comments and Response:

1. Line 320 B. serrata

Response:

Thank you for the careful observation. We have corrected the typographical error in '*B. serrata*' as suggested.

Reviewer #3 Comments and Response:

1. The additional information provided in the Herbal Materials section is somewhat helpful in explaining the origin of the 3 herbal extracts under study here. However, references are still essential to support "their demonstrated bioactivities in gastrointestinal or inflammatory conditions". Likewise, how are these Extracts currently used commercially? In fact, an internet search for 'K Pharms Co., Ltd. of Suwon, Korea' found no company of that name manufacturing herbal extracts.

Response:

We appreciate the verification effort. We would like to clarify that the manufacturer, K Pharms Co., Ltd., was formerly known as Yeskin Co., Ltd. and officially changed its name in 2024. This likely explains the difficulty in the internet search. Regarding the application, we have updated the text to indicate that these extracts are formulated for veterinary health support, and we have added representative citations to the Materials section to support the bioactivity claims(line87-89). Furthermore, the manufacturer is still trying to develop as a therapeutics in calf diarrhea. After licensing from Korean government, these materials will be commercialized.

2. The request for a direct comparison of Extracts A & C would not be difficult, since the data is already included. In fact, this comparison could actually enhance the paper's "mechanistic hypothesis". In a cursory comparison of the results, it appears that Extracts A & C act very similarly except for the data shown in Fig. 2. This may be an important mechanistic finding, based on the 2 additional components of Extract C.

Response:

We agree that a direct comparison between Extract A and Extract C provides valuable context for the mechanistic hypothesis. The distinct antiviral profiles observed in Fig. 2 offer supporting evidence for the functional impact of the differential formulation composition. We have updated the Discussion section (lines 324-325) to explicitly incorporate this comparative analysis.

3. While the manuscript's title is improved without listing the individual extract components, it still doesn't address the potential application of this study. Are these

Extracts currently used in animal disease control? Isn't that why bovine PBMC's were employed? Including more information on the background & current usage of these Extracts in the Materials & Methods, as requested above, may help formulate a better title to promote their veterinary usage.

Response:

We appreciate this constructive suggestion. We have revised the manuscript title to 'Antiviral and Anti-inflammatory Evaluation of Herbal Extracts: Implications for the Management of Calf Diarrheal Diseases' to explicitly highlight the clinical application. This update clarifies the veterinary context and justifies the use of bovine specific models. We tried to reveal the immunomodulatory effects of those materials in bovine. Although we tried to find out appropriate bovine immune cells to conduct our study, we could not find the cell line. Therefore, we decided to use bovine PBMC.

We thank the reviewers for their constructive feedback, which has significantly improved the quality of our work. We hope the revised manuscript is now suitable for acceptance.

Sincerely,
The authors
