# Supplementary material for: Antiviral and anti-inflammatory evaluation of herbal extracts: Implications for the management of calf diarrheal diseases
Source: PLoS One. 2026 Feb 6;21(2):e0342013. doi: 10.1371/journal.pone.0342013 (PMC12880661; doi:10.1371/journal.pone.0342013)
Supplement: Attachment — Submitted filename: Response Letter to Reviewers.docx [file pone.0342013.s005.docx]

Response Letter to Reviewers' Comments

Dear Editor and Reviewers,

We are pleased to resubmit a comprehensively revised and substantially improved manuscript for possible publication in PLOS ONE.

Below we present our detailed responses to each comment from the editor and reviewers. We appreciate the insightful and constructive comments, which have helped us to further improve the manuscript. Each comment has been addressed carefully, and the corresponding revision have been incorporated into the text.

To facilitate your review of our responses and the revised manuscript, we have used the following color-coding approach in our reply letter.

| - Black text | indicates document navigational headings and identifiers. |
| --- | --- |
| - Grey text | represents the original comments from the editor and reviewers. |
| - Purple text | indicates our direct responses to the editor's and reviewers' comments. |

Additionally, we have prepared a comprehensive table of contents to aid navigation and enable quick access to specific sections.

Table of Contents

[Journal Requirements and Response:	2](#_Toc448439466)

[Reviewer #1 Comments and Response:	3](#_Toc1401987739)

[Reviewer #2 Comments and Response:	6](#_Toc1017354489)

[Reviewer #3 Comments and Response:	11](#_Toc412099209)

[Reviewer #4 Response:	13](#_Toc516644088)

# Journal Requirements and Response:

1. Please ensure that your manuscript meets PLOS ONE's style requirements, including those for file naming.

Response:

Thank you for pointing this out. We have carefully reviewed the *PLOS ONE* manuscript preparation and file naming guidelines and revised all submitted files accordingly.

2. Please state what role the funders took in the study.
3. Please note that funding information should not appear in any section or other areas of your manuscript.

Response：

We have updated the funding information as required.

4. When completing the data availability statement of the submission form, you indicated that you will make your data available on acceptance. We strongly recommend all authors decide on a data sharing plan before acceptance, as the process can be lengthy and hold up publication timelines.

Response：

We confirm that all underlying data will be made fully available upon acceptance.

5. Your ethics statement should only appear in the Methods section of your manuscript.
6. Please include captions for your Supporting Information files at the end of your manuscript, and update any in-text citations to match accordingly.

Response：

We have reviewed the manuscript as required.

7. Please review your reference list to ensure that it is complete and correct.

Response：

We have checked the reference list and confirmed that no retracted papers are cited. Four new references were added (now listed as 36, 37, 65 and 66), and the previous reference 57 has been moved to 41 following revisions to the Introduction.

# Reviewer #1 Comments and Response:

1. Line 25 What is Extract C? Specify the composition.

Response:

Thank you for your comment. To avoid confusion in the Abstract, we have revised the wording to clarify that the three formulations used in this study (lines 15-17) are 'three distinct herbal extracts (designated Extracts A, B, and C)'. This explicitly defines Extract C as requested.

2. Line 28 Please use a different word rather than Sophisticated

Response:

We agree and have replaced 'sophisticated' with 'complex' in the revised manuscript, as requested (line 24).

3. Line 185 The conclusion of'good biological safety,' which is based solely on the inability of the extracts to suppress the growth of immortalized cells, appears highly presumptive.

Response:

We agree that the term 'good biological safety' is not fully supported by cell viability data alone. Therefore, we have revised the wording to 'did not show obvious cytotoxicity' to more accurately reflect the results (lines 208-209).

4. Lines 287, 372 and elsewhere in the manuscript: The tested extracts exhibited an antioxidant effect, as evidenced by the suppression of NO production; however, they also showed a pro-inflammatory effect, since some of them increased the levels of pro-inflammatory cytokines IL-6, IFNg and TNF-α. Therefore, the claim of an anti-inflammatory effect should be reconsidered.

Response:

Thank you for this critical suggestion. We agree that our data demonstrates a complex, dual effect and that our original claim of a simple 'anti-inflammatory' effect was doubtful. We have addressed this point by revising the text throughout the manuscript (including the Abstract, Discussion, and Conclusion). We have replaced the term 'anti-inflammatory' with the more precise description of 'complex immunomodulatory effects'.

5. Line 288. An evaluation of T cell subsets was not performed in this work, please rephrase.

Response:

We have rephrased this sentence (and similar instances) to refer to 'T helper cell-associated responses' by revising the text throughout the manuscript.

6. Discussion. The Discussion should focus on interpreting the findings rather than repeating the results or referencing the figures again

Response:

Thank you for the comment. We have revised the Discussion to focus on interpretation as reviewer recommended.

7. Fig. 2 The authors should discuss the discrepancy between the upregulation of iNOS mRNA expression and the significant reduction in NO production observed following treatment with Extract C.

Response:

Thank you for this insightful comment. We agree that the divergence between the upregulation of iNOS mRNA and the reduction in NO production observed with Extract C requires clarification.

We have expanded the Discussion to address this point. As now noted in the revised text, iNOS transcription does not always correlate with enzymatic activity or NO output, and several studies have reported similar dissociations in natural-product models. Extract C may suppress NO production through post-transcriptional regulation such as reduced translation or enhanced protein turnover) or by interfering with iNOS catalytic activity, rather than blocking iNOS gene induction. We also have added relevant references (Hung et al., 2019; Tanemoto et al., 2015) to support this interpretation.

8. Line 238: FOXP3 is considered a T cell specific marker, particularly of Tregs, it is unlikely that RAW264.7 express FOXP3 mRNA under normal conditions. Please check the specificity of your primers. Wouldn't it be more appropriate to assess FoxP3 expression following extract treatment in PBMC fraction?

Response:

Thank you for this important comment. We agree that FOXP3 is a marker associated with regulatory T cells and is not expected to be expressed in RAW264.7 macrophages. In line with your suggestion, we have removed FOXP3 data for RAW264.7 cells from the figures and the text. The analysis of FOXP3 is now limited to PBMC samples, where T cell related responses can be evaluated in an appropriate biological context. We also rechecked the FOXP3 primers and confirmed that they produce a single specific product in PBMC samples (Table 3).

Reference

Hung YL, Wang SC, Suzuki K, Fang SH, Chen CS, Cheng WC, et al. Bavachin attenuates LPS-induced inflammatory response and inhibits the activation of NLRP3 inflammasome in macrophages. Phytomedicine. 2019;59:152785. Epub 20181210. doi: 10.1016/j.phymed.2018.12.008. PubMed PMID: 31009850.

Tanemoto R, Okuyama T, Matsuo H, Okumura T, Ikeya Y, Nishizawa M. The constituents of licorice (Glycyrrhiza uralensis) differentially suppress nitric oxide production in interleukin-1β-treated hepatocytes. Biochemistry and biophysics reports. 2015;2:153-9. doi: 10.1016/j.bbrep.2015.06.004.

# Reviewer #2 Comments and Response:

Introduction:

1. The current study is in vitro but the introduction focused on calf diarrhea rather than discussing the components of the different herbal combinations used in the study. I would recommend rewriting the introduction to focus on the different active compounds in the herbal combinations and the previous studies that support them.

Response:

Thank you for the helpful suggestion. The Introduction has been reorganized to focus more directly on the active herbal components and the supporting literature (lines 61-72).

2. The objectives are vague and need to be rewritten to reflect the current study objectives and have to clarify that the evaluation will be done in vitro.

Response:

In line with this revision, we have also rewritten the study objectives so that they are more precise and clearly indicate that all evaluations were performed *in vitro*. The revised objectives appear in lines (lines 73-76).

3. Lines 70-72:'The goal of this study was to identify a safe and effective candidate that offers a natural therapeutic option for viral diarrhea in calves and aligns with the 'One Health'concept, which emphasizes maintaining ecosystem balance and human well-being by safeguarding animal health'. With the current study design (in vitro), the authors can't identify the safety or evaluate the effectiveness against viral diarrhea in calves, which will require an in vivo study, which is not the case for the current study.

Response:

We agree that the original sentence went beyond what can be supported by an in vitro study. Since our work does not include any *in vivo* experiments, it cannot address safety or clinical effectiveness in calves. To avoid giving an impression that exceeds the scope of the study, we have removed the sentence in lines 70–72 and revised the objectives so that they clearly reflect the in vitro nature of the work (lines73-76). We appreciate the reviewer's careful reading.

Materials and methods

4. There is no clear timeline for the current study.

Response:

We have added the specific experimental time points to clarify the study timeline of each treatment.

5. There is no mention of where that work was done (which lab) and who did the lab work.

Response:

Thank you for the comment. We have added a statement in the Materials and Methods section specifying where the laboratory work was carried out and by whom (lines 80-81).

6. Line 156 'Table 2Error! Reference source not found..' what does that mean?

Response:

We sincerely apologize for this formatting oversight. This text appeared due to a broken cross-reference field in the manuscript file during the conversion process.

7. For the cytotoxicity assay lines 107-121. It is not clear how many wells were assigned for control and each extract.

Response:

We have added the number of replicate wells used for each control and treatment group; each condition was tested in four technical replicates (line 122).

8. Why the PC was only used for the PBMCs in the cytotoxicity testing?

Response:

Thank you for the comment. In RAW 264.7 cells, 1 μg/mL LPS is a widely used standard concentration for macrophage activation and is well established in the literature as non-cytotoxic(Facchin et al., 2022). Because this dose functions as an inflammatory inducer rather than a viability-reducing stimulus, including it as a separate condition in the cytotoxicity assay would not provide meaningful information.

In contrast, LPS is used less frequently in bovine PBMC systems, and in this study an LPS-treated group was required as a positive reference for the downstream immunoactivity assays. For this reason, we confirmed its safety in PBMCs by including it in the viability test, whereas a separate LPS condition was not needed in the RAW 264.7 cytotoxicity assay.

9. For the testing of antiviral effect on BCoV and BRV lines 129-141, it is not clear how many cells were assigned for controls and each extract.

Response:

We have added the number of wells used per condition and confirmed that the assay was performed in three independent repeats (lines 145, 160).

10. For testing of anti-inflammatory effects onRAW264.7 cells lines, 145-156, it is not clear how many cells were assigned for controls and each extract.

Response:

The Methods now specify the wells assigned to each group, and all assays were carried out in three independent experiments (lines 165, 177).

11. For testing of Immunomodulatory effects on RAW 264.7 cells and bovine PBMCs, lines 160-167, it is not clear how many cells were assigned for controls and each extract.

Response:

We have clarified the well numbers for each condition, and these assays were also repeated independently three times (lines 182, 189).

12. For the primers that referenced as this study, there is no explanation of how the authors decided or designed these primers. Please add a section explaining the procedures that were followed by the authors to design these primers.

Response:

Thank you for this suggestion. We have added a detailed description of the primer design procedure in the Methods section (lines 154-157). We clarified that the primers were designed using NCBI Primer-BLAST based on GenBank sequences, and their specificity was validated both in silico (BLAST) and experimentally (melting curve analysis). Please refer to the revised text for details.

13. Statistical analysis, Was there any pairwise comparisons done? If yes what are they? If not why not?

Response:

Yes, pairwise comparisons were performed. Statistical significance was evaluated using one-way ANOVA followed by Dunnett's post-hoc test, comparing each extract-treated group with the corresponding control group. This information has been added to the Statistical analysis section (lines 198-199) and clarified in the figure legends.

Results

14. Line 182: '1Error! Reference source not found.,' what does that mean?

Response:

We sincerely apologize for this formatting oversight. We have corrected the formatting error in the revised manuscript.

15. The colors for figures 4-6 are not clear, I would recommend using colors that can be easily distinguished.

Response:

Thank you for the comment. Figures 4-6 have been updated with clearer and more distinguishable colors.

Discussion

16. Lines 288-290: 'Collectively, these findings support a new comprehensive treatment strategy for calf viral diarrhea through the synergistic effect of multiple targets and provide new ideas for prevention and treatment.' That statement is overestimated the study outcomes, please be more specific to the study outcomes as the current study is only in vitro study.

Response:

We have removed the overstated sentence from the original version and revised the text so that the conclusions and objective reflect only the scope of the *in vitro* findings.

Reference

Facchin BM, dos Reis GO, Vieira GN, Mohr ETB, da Rosa JS, Kretzer IF, et al. Inflammatory biomarkers on an LPS-induced RAW 264.7 cell model: a systematic review and meta-analysis. Inflammation Research. 2022;71(7):741-58. doi: 10.1007/s00011-022-01584-0.

# Reviewer #3 Comments and Response:

1. First, it would be helpful if the authors could provide a reference as well as some sort of explanation as to how these extracts were prepared in the Herbal Materials section. It would also be good to know why these Extracts were formulated as they are, &/or why these particular extracts were selected for study.

Response:

Thank you for this comment. We have revised the Herbal Materials section to include additional information on the source, composition, extraction description, and selection rationale of the three extracts. A brief explanation has been added noting that the extracts were standardized products provided by K Pharms Co., Ltd., produced using standard solvent extraction procedures, and selected based on their traditional use in gastrointestinal or inflammatory conditions (lines 87-91).

2. Second, since Extract C is apparently the same as Extract A but with 2 additional components, it would be extremely valuable to directly compare any differences observed between the responses elicited with & without these 2 components. My apologies if any such comparisons in this regard were missed.

Response:

We thank the reviewer for this insightful comment. The present study was designed to evaluate each extract relative to its control group; therefore, no statistical comparison was performed between Extract A and Extract C. The discussion referring to compositional differences was intended only as a mechanistic hypothesis rather than a direct comparison between extracts.

3. Lastly, the manuscript's title may also be somewhat misleading in this respect. It may imply to readers that extracts from each of these sources are being assessed individually. Something less specific may be better, such as; 'Anti-viral, anti-inflammatory, and immunomodulatory effects of herbal extracts in a cellular model of bovine diarrhea', although not all cells were bovine so this may not be a perfect option.

Response:

Thank you for this helpful comment. We agree that the original title may give the impression that the herbal sources were evaluated individually. To avoid this potential misunderstanding and to more accurately reflect the study design, we have revised the title.

The new title is:

In vitro antiviral and immunomodulatory activities of three herbal extract formulations

4. Correct referencing errors flagged on lines 156 & 182.

Response:

We sincerely apologize for this formatting oversight. This text appeared due to a broken cross-reference field in the manuscript file during the conversion process.

# Reviewer #4 Response:

Thank you very much for your encouraging feedback on our manuscript. We sincerely appreciate your kind comments regarding the quality of our research and the writing. Your positive endorsement has been instrumental in validating our work, and we are grateful for your time and effort in reviewing it.

We believe that the revised manuscript has been substantially improved and now meets the standards of the journal. We hope that our responses address the reviewers' comments satisfactorily, and we would be pleased to consider any further suggestions.

Best regards,

The authors
